# An endothelial SOX18–mevalonate pathway axis enables repurposing of statins for infantile hemangioma

**DOI:** 10.1172/JCI179782

**Published:** 2025-02-25

**Authors:** Annegret Holm, Matthew S. Graus, Jill Wylie-Sears, Jerry Wei Heng Tan, Maya Alvarez-Harmon, Luke Borgelt, Sana Nasim, Long Chung, Ashish Jain, Mingwei Sun, Liang Sun, Pascal Brouillard, Ramrada Lekwuttikarn, Yanfei Qi, Joyce Teng, Miikka Vikkula, Harry Kozakewich, John B. Mulliken, Mathias Francois, Joyce Bischoff

**Affiliations:** 1 Vascular Biology Program, Department of Surgery, Boston Children’s Hospital, Harvard Medical School, Boston, Massachusetts, USA.; 2 The David Richmond Laboratory for Cardiovascular Development: Gene Regulation and Editing, Centenary Institute, University of Sydney, Camperdown, New South Wales, Australia.; 3 Research Computing, Information Technology, Boston Children’s Hospital, Boston, Massachusetts, USA.; 4 Human Molecular Genetics, de Duve Institute, University of Louvain, VASCERN-VASCA European Reference Center, Brussels, Belgium.; 5 Department of Dermatology, Lucile Packard Children’s Hospital at the Stanford University School of Medicine, Palo Alto, California, USA.; 6 WELBIO Department, WEL Research Institute, Wavre, Belgium.; 7 Department of Pathology, Boston Children’s Hospital, Harvard Medical School, Boston, Massachusetts, USA.; 8 Department of Plastic and Oral Surgery, Boston Children’s Hospital; Department of Surgery, Harvard Medical School; Boston, Massachusetts, USA.; 9 School of Biomedical Sciences, University of Sydney, Camperdown, New South Wales, Australia.

**Keywords:** Angiogenesis, Vascular biology, Cholesterol, Endothelial cells, Transcription

## Abstract

Infantile hemangioma (IH) is the most common tumor in children and a paradigm for pathological vasculogenesis, angiogenesis, and regression. Propranolol, the mainstay of treatment, inhibits IH vessel formation via a β-adrenergic receptor-independent off-target effect of its R(+) enantiomer on endothelial SOX18 - a member of the SOX (SRY-related HMG-box) family of transcription factors. Transcriptomic profiling of patient-derived hemangioma stem cells uncovered the mevalonate pathway (MVP) as a target of R(+) propranolol. Loss and gain of function of SOX18 confirmed it is both necessary and sufficient for R(+) propranolol suppression of the MVP, including regulation of sterol regulatory element–binding protein 2 (SREBP2) and the rate-limiting enzyme HMG-CoA reductase (HMGCR). A biological relevance of the endothelial SOX18-MVP axis in IH patient tissue was demonstrated by nuclear colocalization of SOX18 and SREBP2. Functional validation in a preclinical IH xenograft model revealed that statins — competitive inhibitors of HMGCR — efficiently suppress IH vessel formation. We propose an endothelial SOX18-MVP axis as a central regulator of IH pathogenesis and suggest statin repurposing to treat IH. The pleiotropic effects of R(+) propranolol and statins along the SOX18-MVP axis to disable an endothelial cell–specific program may have therapeutic implications for other vascular disease entities involving pathological vasculogenesis and angiogenesis.

## Abstract 2

A SOX18-mevalonate pathway axis controls vascular overgrowth in infantile hemangioma revealing potential for statin therapy in this common vascular tumor.

## Graphical abstract

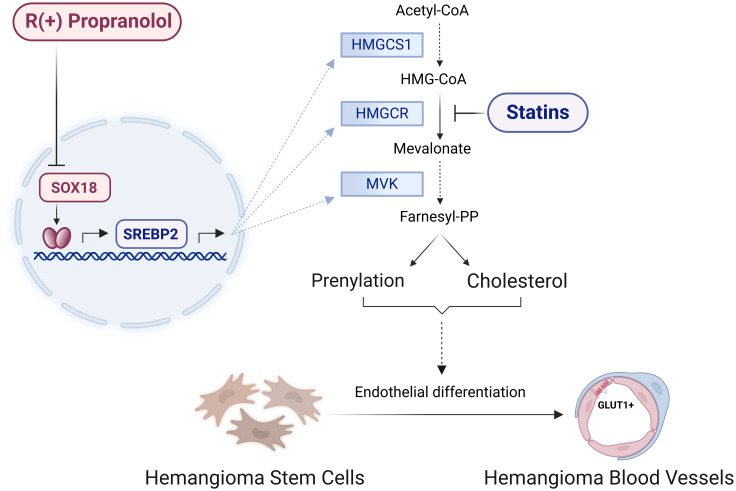

## Introduction

Infantile hemangioma (IH) is a benign vascular tumor of infancy with an incidence of 2%–10%. It predominantly occurs in female and premature infants of European descent. IH arises postnatally at 2–7 weeks of age with rapid neovascularization during the proliferating phase, which can continue for 4–18 months of age. A spontaneous and gradual involuting phase follows, spanning 3–9 years. A subset of IH — termed rebounding or regrowing IH — does not undergo complete involution and may regrow after treatment discontinuation. The underlying cellular mechanism of IH formation is the differentiation of multipotent hemangioma stem cells (HemSCs) into hemangioma endothelial cells (HemECs) and hemangioma pericytes to form neovessels (vasculogenesis), concurrent with cellular proliferation (1–4). For most children, IH poses no serious risk; however, 10%–15% of lesions require treatment to prevent sequelae such as disfigurement, functional impairment including vision loss and airway obstruction, consumptive hypothyroidism, and high-output cardiac failure (5). Propranolol was discovered serendipitously to be effective for IH; it is currently the only FDA-approved drug for IH (6). Despite successful repurposing for IH, propranolol is associated with β-adrenergic side effects including hypotension, bradycardia, peripheral vasospasm, bronchospasm, hypoglycemia and seizures, sleep disturbance, and potentially adverse neurocognitive outcomes (6–10). The complete response rate to propranolol is reported to be 60% (6). These safety and efficacy concerns underscore the need for additional treatments for IH. Therapeutic avenues have remained limited because of a lack of information on the molecular basis of propranolol’s mode of action in IH.

Propranolol acts as a nonselective antagonist of β_1_- and β_2_-adrenergic receptor GPCRs; it is a 1:1 mixture of S(–) and R(+) enantiomers. The S(–) enantiomer is a potent antagonist of β_1_- and β_2_-adrenergic receptors, while the R(+) enantiomer is largely devoid of beta blocker activity (11). This provided an opportunity to identify an R(+) propranolol–dependent pathway in HemSCs (12, 13). HemSCs are isolated from proliferating-phase IH specimens, are mesenchymal in nature, exhibit multilineage differentiation potential, and have been shown to recapitulate hemangiogenesis in nude mice (2). R(+) propranolol inhibits HemSC endothelial differentiation in vitro and HemSC vasculogenesis in a preclinical IH model (12, 13). Furthermore, we established that R(+) propranolol interferes directly with the activity of endothelial transcription factor SOX18 - a member of the SOX (SRY-related HMG-box) family of transcription factors. In addition to propranolol, pharmacological interference with SOX18 is achieved through use of the small-molecule inhibitor Sm4 (14), which provided further validation of the role of SOX18 in IH (12, 13). These findings have led to the repurposing of propranolol in patients with hypotrichosis–lymphedema–telangiectasia–renal defect syndrome (HLTRS), a rare vascular disease caused by a dominant-negative truncating mutation in SOX18 (15). These observations provided evidence for the pharmacogenetic interaction between SOX18 and propranolol in a vascular disease (12). In summary, we identified a SOX18-dependent inhibition of HemSC endothelial differentiation and vessel formation in vivo as the molecular basis of propranolol-mediated inhibition of vessel formation in IH.

SOX18 is a master transcriptional regulator of vascular development and endothelial specification and is expressed in nascent blood and lymphatic endothelium as well as in endothelial progenitor cells (16). It plays fundamental roles in arterial specification, lymphangiogenesis, and angiogenesis (17) and in tumor angiogenesis (18). Its known role in instructing the molecular program of endothelial specification and differentiation prompted us to investigate the role of SOX18 in the context of HemSC endothelial differentiation.

In this study, we set out to identify genes whose expression in differentiating HemSCs is altered by R(+) propranolol–mediated inhibition of SOX18. We discovered that R(+) propranolol rapidly downregulates transcripts encoding enzymes in the mevalonate pathway (MVP) in a SOX18-dependent manner. The MVP is central to cholesterol and isoprenoid biosynthesis and is controlled by the rate-limiting enzyme HMG-CoA reductase (HMGCR) (19), which produces mevalonate. A critical regulator of MVP genes is the transcription factor sterol regulatory element–binding protein 2 (SREBP2) (20).

We show that statins, competitive HMGCR inhibitors, potently reduce blood vessel formation in a preclinical IH model. Statins are widely prescribed to reduce LDL-cholesterol in patients at risk for cardiovascular disease (21). As a consequence of inhibiting the MVP, statins increase LDL receptor expression, thereby enhancing clearance of LDL-cholesterol from the bloodstream. Here, we uncover a molecular relationship between the endothelial cell–specific transcription factor SOX18 and the MVP. We demonstrate that R(+) propranolol downregulates MVP genes in HemSCs during their endothelial differentiation. In vitro, using loss and gain of function–based approaches, we show that SOX18 is necessary and sufficient for R(+) propranolol–mediated regulation of the MVP. We propose that SOX18 may act as a rheostat of the MVP in pathological endothelium, and we determine that this axis is critical to IH disease progression. Blocking the MVP with statins inhibits HemSC endothelial differentiation and vessel formation, suggesting that statins could be repurposed to treat IH.

## Results

### R(+) propranolol–induced SOX18 inhibition reduces MVP gene expression during HemSC endothelial differentiation.

To identify the downstream targets of R(+) propranolol in HemSC to HemEC differentiation, we performed bulk RNA-Seq of HemSCs isolated from 6 different IH specimens. Table 1 provides an overview of patient samples used in respective experiments. HemSCs were induced to undergo endothelial differentiation for 6 days (2) and then treated with or without R(+) propranolol (20 μM) for 2 hours (Supplemental Figure 1.1; supplemental material available online with this article; https://doi.org/10.1172/JCI179782DS1). HemSCs at day 4 of endothelial differentiation, prior to onset of endothelial marker expression, were treated with R(+) propranolol and analyzed as well. The timing and dose of R(+) propranolol were determined by previously observed downregulation of *NOTCH1* expression, a SOX18 transcriptional target (13). Endothelial differentiation of HemSCs was validated by increased relative gene expression in SOX18 and VE-cadherin over the course of 6 days (Supplemental Figure 1.2).

**Figure 1:**
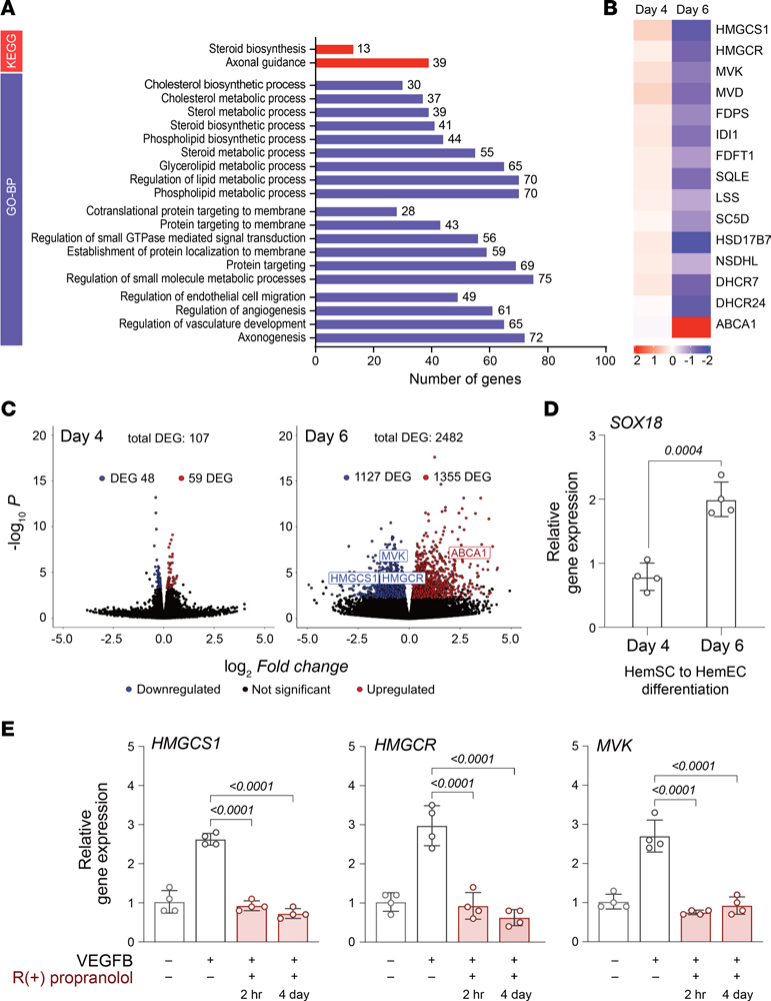
R(+) propranolol reduces MVP transcripts in IH-derived HemSCs undergoing endothelial differentiation. HemSCs in **A**–**C** were treated with or without R(+) propranolol for 2 hours before RNA isolation. (**A**) KEGG pathway analysis and Gene Ontology Biological Processes (GO-BP) analysis of bulk RNA-Seq data from HemSCs treated on day 6 of endothelial differentiation (*n* = 6 biological replicates) with or without R(+) propranolol, showing the number of differentially expressed genes (DEGs), defined as log_2_ fold change greater than 1 and adjusted *P* less than 0.05. (**B**) Heatmap of differentially expressed MVP genes at day 4 and day 6. (**C**) Volcano plots of DEGs on day 4 and day 6 of HemSC to HemEC differentiation (–log_10_ for adjusted *P* value). (**D**) *SOX18* mRNA levels on day 4 and day 6 (*n* = 3 biological replicates; 1 of the biological replicates was analyzed in 2 independent experiments, yielding *n* = 4 data points). (**E**) HemSCs induced to undergo endothelial differentiation were treated with or without R(+) propranolol for 2 hours on day 6 or treated continuously from day 2 to day 6. qPCR analyses for *HMGCS1*, *HMGCR*, and *MVK* (*n* = 3 biological replicates; 1 of the biological replicates was analyzed in 2 independent experiments, yielding *n* = 4 data points). *P* values were calculated using a 2-tailed, unpaired *t* test (**D**) and by a 1-way ANOVA multiple-comparison test with Šidák correction (**E**). Data are shown as the mean ± SD.

The Kyoto Encyclopedia of Genes and Genomes (KEGG) analysis identified steroid biosynthesis as differentially affected on day 6. This was confirmed by Gene Ontology analysis of the subontology “biological processes,” with most of the top terms related to cholesterol or isoprenoid biosynthesis and processes involved in angiogenesis (Figure 1A and Supplemental Figure 2). Figure 1B shows a heatmap of differentially expressed MVP genes as well as genes in cholesterol and isoprenoid biosynthesis on day 4 and day 6. Gene-by-gene analysis revealed that R(+) propranolol treatment on day 6 significantly reduced transcripts encoding several enzymes of the MVP, including the rate-limiting enzyme HMGCR as well as HMGCS1 and mevalonate kinase (MVK), while few changes were seen at day 4 (Figure 1, B and C, and Supplemental Figure 2). Notably, ABCA1 — a negative regulator of the MVP (22) — was upregulated at day 6. Overall, 107 genes in HemSCs were differentially expressed at day 4 versus 2,482 genes at day 6 of differentiation (Figure 1C). Differential expression was defined as log_2_ fold change greater than 1 and adjusted *P* value less than 0.05. Comparative analysis of the time points defines a window for SOX18 inhibition using R(+) propranolol, supported by the lack of downregulation of MVP transcripts at day 4 of differentiation, when relatively low endothelial marker and SOX18 expression was seen (Figure 1D, Supplemental Figure 1.2, and Supplemental Figure 2). The significant increase in *SOX18* mRNA from day 4 to day 6 of endothelial differentiation is consistent with the onset of MVP gene sensitivity to R(+) propranolol at day 6 (Figure 1D). Downregulation of *HMGCS1*, *HMGCR*, and *MVK* was confirmed by quantitative PCR (qPCR) in cells treated with R(+) propranolol for 2 hours or continuously for 4 days of the endothelial differentiation protocol (Figure 1E).

**Figure 2:**
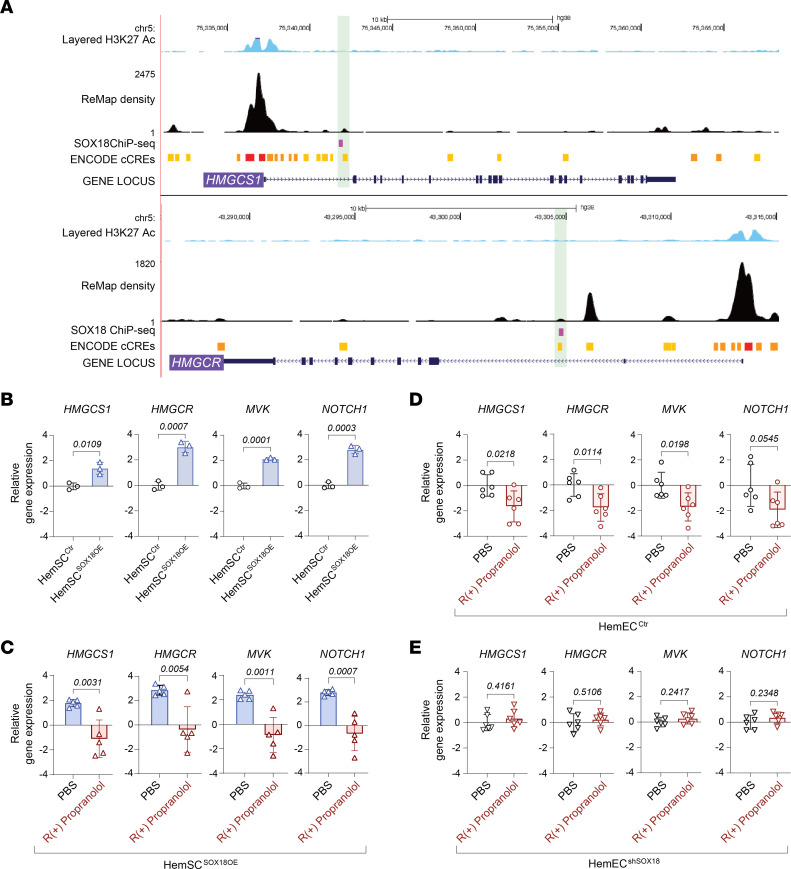
SOX18 fine-tunes endothelial MVP gene expression. (**A**) ChIP-Seq dataset in HUVECs identifies SOX18-binding sites within the *HMGCS1* and *HMGCR* gene loci. (**B**) Lentiviral SOX18 overexpression in undifferentiated HemSCs (HemSC^SOX18OE^) versus control HemSCs (HemSC^Ctr^). RNA was analyzed for *HMGCS1*, *HMGCR*, *MVK*, and *NOTCH1* by qPCR (*n* = 3 independent experiments). (**C**) Treatment of HemSC^SOX18OE^ with or without R(+) propranolol for 24 hours (*n* = 5 independent experiments). (**D**) Treatment of IH-derived control HemECs with R(+) propranolol for 24 hours. RNA was analyzed for *HMGCS1*, *HMGCR*, *MVK*, and *NOTCH1* by qPCR. (**E**) HemECs with lentiviral knockdown of SOX18 (HemEC^shSOX18^) were treated with R(+) propranolol for 24 hours followed by qPCR analyses (*n* = 3 biological replicates, performed in 2 independent experiments, yielding *n* = 6 data points; lentiviral knockdown of SOX18 was performed twice in each of the 3 HemEC lines with an efficiency cutoff of >70%). *P* values were calculated using a 2-tailed, unpaired *t* test (**B**–**E**). Data are shown as the mean ± SD.

To further elucidate the effect of SOX18 inhibition on the MVP, we investigated SOX18-binding locations in the genome using a publicly available ChIP-Seq dataset in HUVECs (23). We discovered SOX18-binding sites within the *HMGCS1* and *HMGCR* gene loci that corresponded with ENCODE-defined *cis*-regulatory elements (Figure 2A). R(+) propranolol–induced changes in gene expression, combined with the identification of SOX18-binding sites in regulatory regions of *HMGCS1* and *HMGCR*, support the possibility that interference of SOX18 activity may perturb transcriptional regulation of critical genes along the MVP pathway.

### SOX18 gain and loss of function confirm its role as an endothelial fine-tuner of MVP genes.

To interrogate the role of SOX18 in regulating the MVP, we used gain of function (lentiviral overexpression) and gene depletion (shRNA knockdown) in HemSCs and HemECs, respectively. SOX18 overexpression (OE) in undifferentiated HemSCs resulted in a significant increase in the mRNA levels of *HMGCS1*, *HMGCR*, *MVK*, and *NOTCH1* (Figure 2B). The known SOX18 target *NOTCH1* served as a positive control for SOX18 transcriptional activity. R(+) propranolol reduced expression of all 4 genes in HemSC^SOX18OE^ (Figure 2C). This demonstrates that SOX18 is sufficient to drive the MVP in HemSCs. We next knocked down SOX18 in HemECs that express high levels of SOX18. mRNA levels of *HMGCS1*, *HMGCR*, *MVK*, and *NOTCH1* in control HemECs were significantly reduced by R(+) propranolol (Figure 2D), as seen in HemSCs undergoing endothelial differentiation (Figure 1E). SOX18 knockdown abolished the R(+) propranolol effect on expression of *HMGCS1*, *HMGCR*, *MVK*, and *NOTCH1* in HemEC^shSOX18^ (Figure 2E). This demonstrates that SOX18 is needed to increase MVP gene expression in HemECs. In summary, these experiments demonstrate via genetic manipulation that SOX18 is both necessary and sufficient to regulate the transcription of MVP genes and to mediate the pharmacological effect of R(+) propranolol on the MVP.

### SOX18 positively regulates MVP biosynthetic output in human endothelial cells.

**Figure 3:**
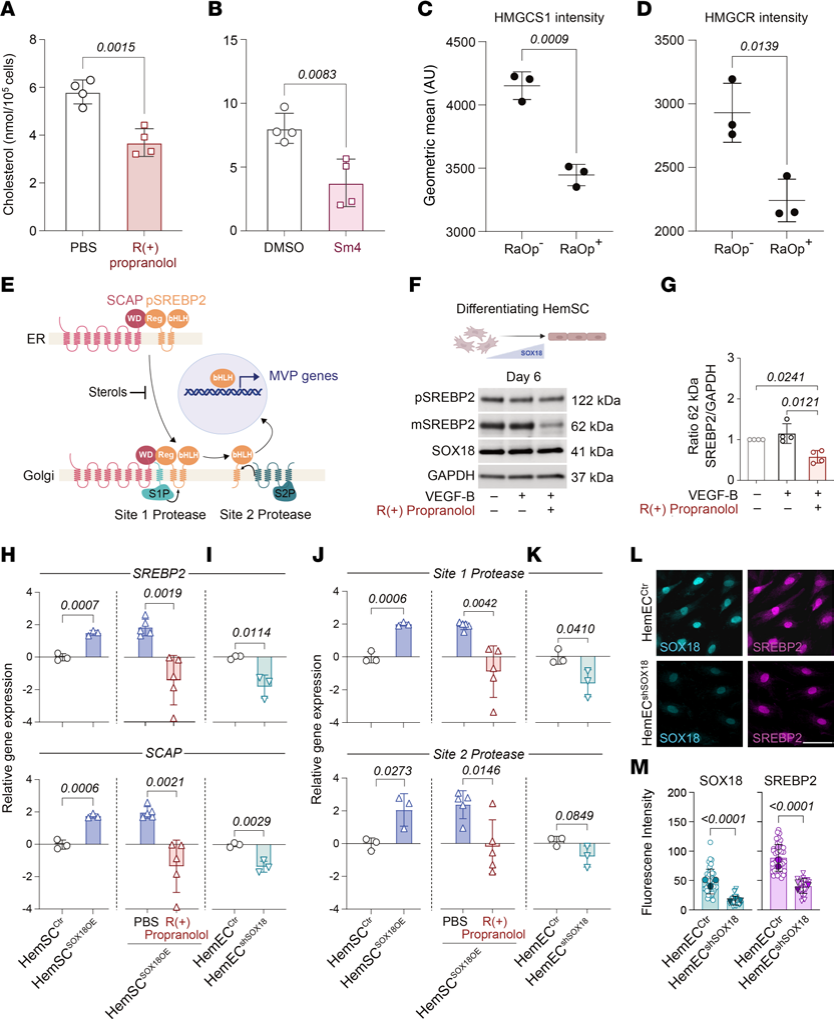
Biosynthetic output of the MVP is mediated via a SOX18-/SREBP2-dependent mechanism. (**A** and **B**) HUVECs were incubated with MBCD for 16 hours, followed by treatment with or without R(+) propranolol or with or without Sm4 for 16 hours. Endogenous cholesterol levels were measured by mass spectrometry (*n* = 3 biological replicates). (**C** and **D**) Overexpression of *ragged-opossum* (*RaOp*) in HUVECs followed by immunofluorescence staining of HMGCS1 and HMGCR (*n* = 3 biological replicates). *P* values were calculated using a 2-tailed, unpaired *t* test (**A** and **B**) and by a 1-way ANOVA multiple-comparison test with Tukey’s correction (**C** and **D**). Data are shown as the mean ± SD. (**E**) Schematic of SREBP2 maturation by SCAP, site 1 protease (S1P), and S2P to produce the bHLH domain that translocates to the nucleus. (**F** and **G**) HemSCs undergoing endothelial differentiation for 6 days were treated for 2 hours with or without R(+) propranolol, lysed, and analyzed by WB with anti-SREBP2, anti-SOX18, and anti-GAPDH (*n* = 3 biological replicates; 1 of the biological replicates was analyzed in 2 independent experiments, yielding *n* = 4 data points). (**H** and **J**) Relative gene expression of *SREBP2*, *SCAP*, *S1P*, and *S2P* in HemSC^Ctr^ versus HemSC^SOX18OE^, with HemSC^SOX18OE^ treated with or without R(+) propranolol for 24 hours. (**I** and **K**) Relative gene expression of *SREBP2*, *SCAP*, *S1P*, and *S2P* in HemEC^Ctr^ versus HemEC^shSOX18^. (**L** and **M**) Immunofluorescence staining of SOX18 and SREBP2 in HemEC^shSOX18^ versus HemEC^Ctr^ (*n* = 3 biological replicates). Scale bar: 25 μm. *P* values were calculated using a 2-tailed, unpaired *t* test (**A**–**D** and **H**–**M**) and a 1-way ANOVA multiple-comparison test with Šidák correction (**F** and **G**). Data represent sample sizes *n* = 4 (**A** and **B**) and *n* = 3 (**C**, **D**, **I**, **K**, and **M**) biological replicates; *n* = 3 biological replicates with 1 of the biological replicates analyzed in 2 independent experiments yielding *n* = 4 data points (**F** and **G**); and *n* = 3–5 independent experiments (**H** and **J**). Data are shown as the mean ± SD.

To validate the role of SOX18 in regulating genes in the MVP, we tested whether disruption of SOX18 activity with R(+) propranolol or the SOX18 small-molecule inhibitor Sm4 (14) would have an effect on cholesterol biosynthesis in HUVECs. First, HUVECs were depleted of cholesterol by incubation with methyl β-cyclodextrin (MBCD) and treated with or without R(+) propranolol or with or without Sm4 for 16 hours, allowing for new cholesterol synthesis to take place, which was then measured by targeted mass spectrometry. Endogenous cholesterol levels were significantly reduced in R(+) propranolol– and Sm4-treated HUVECs, consistent with SOX18-mediated downregulation of MVP gene expression (Figure 3, A and B). We also tested the effect of an overexpressed, dominant-negative version of SOX18 (*ragged-opossum* [*RaOp*]) that is known to disrupt SOX18 activity (24). Using FACS as a readout, we determined that expression of SOX18*^RaOp^* decreased HMGCS1 and HMGCR protein levels compared with its wild-type control on a cell population scale (Figure 3, C and D, and Supplemental Figure 3.1). This indicates an effect on 2 key MVP enzymes that control this pathway. Both experiments demonstrate that SOX18 positively regulates cholesterol biosynthesis in an endothelial context.

### R(+) propranolol mode of action on the MVP is mediated via a SOX18-/SREBP2-dependent mechanism.

SREBP2 is the master transcriptional regulator of MVP genes and is ubiquitously expressed (20). Based on the above findings, we posit that in HemSCs undergoing endothelial differentiation, SOX18 coordinates with SREBP2. To test this, we analyzed the effect of R(+) propranolol on levels of the precursor SREBP2 (122 kDa), which resides in the endoplasmic reticulum, as well as mature SREBP2 (62 kDa) — the basic helix loop helix (bHLH) leucine zipper domain (20, 25). When endogenous cholesterol levels drop, the inactive 122 kDa precursor is transported to the Golgi and proteolytically cleaved to release the active 62 kDa mature form. The latter translocates into the nucleus to activate transcription of MVP genes (Figure 3E). We hypothesized that R(+) propranolol would reduce 62 kDa SREBP2 in HemSCs undergoing endothelial differentiation. HemSCs (*n* = 4) were treated for 2 hours with or without R(+) propranolol on day 6 of endothelial differentiation. Cell lysates analyzed by Western blot (WB) showed that the R(+) propranolol treatment decreased mature 62 kDa SREBP2 (Figure 3, F and G). Rapid turnover of mature SREBP2 within 4 hours has been demonstrated (26). The reduced levels of 62 kDa SREBP2 upon R(+) propranolol treatment are consistent with the bulk RNA-Seq and qPCR data and suggest that SOX18 increases SREBP2 maturation, thereby influencing the MVP output. The anti–human SREBP2 antibody used to detect precursor and mature forms of SREBP2 was validated in HemSCs depleted of cholesterol by MBCD (Supplemental Figure 3.2).

We next assessed SOX18-dependence of the R(+) propranolol–mediated effects on SREBP2 in gain-of-function and gene depletion experiments as in Figure 3, H–K. We assessed the effect of SOX18 overexpression in HemSCs on SREBP2, its chaperone sterol regulatory element–binding protein cleavage–activating protein (SCAP), and its cleavage proteins site 1 protease (S1P) and site 2 protease (S2P) (20). Indeed, overexpressed SOX18 in HemSCs resulted in significantly increased *SREBP2*, *SCAP*, *S1P*, and *S2P* mRNA levels, which was abolished by R(+) propranolol (Figure 3, H and J). Knockdown of SOX18 in HemECs had the opposite effect: mRNA levels of *SREBP2*, *SCAP*, and *S1P* were significantly reduced; *S2P* was reduced but not significantly (Figure 3, I and K). Immunofluorescence staining of control HemECs and HemEC^shSOX18^ confirmed the efficient knockdown of SOX18 and a corresponding decrease in SREBP2 (Figure 3, L and M), consistent with the reduced SREBP2 transcript level in the HemEC^shSOX18^ (Figure 3I). WB analyses of SREBP2 are consistent with these results (Supplemental Figure 3.3 and 3.4). SOX18 overexpression in undifferentiated HemSCs resulted in significantly increased 62 kDa SREBP2 (Supplemental Figure 3.3). In control HemECs, an increase in 62 kDa of SREBP2 was observed upon R(+) propranolol treatment, likely due to feedback mechanisms over the 24-hour treatment period (26). Importantly, SOX18 knockdown in HemECs abolished the effect of R(+) propranolol on levels of the 62 kDa SREBP2 (Supplemental Figure 3.4). Together, these data suggest that SOX18 increases transcript levels of SREBP2 itself and the genes needed for processing SREBP2 to its active form. We speculate that SOX18 may provide a boost to isoprenoid biosynthesis in differentiating, nascent endothelium by increasing expression of *HMGCS1* and *HMGCR*, as suggested in Figure 2A, and *SREBP2*, *SCAP*, *S1P*, and *S2P*, as shown.

### Nuclear SOX18 and SREBP2 in proliferating-phase and regrowing IH indicate active MVP.

**Figure 4:**
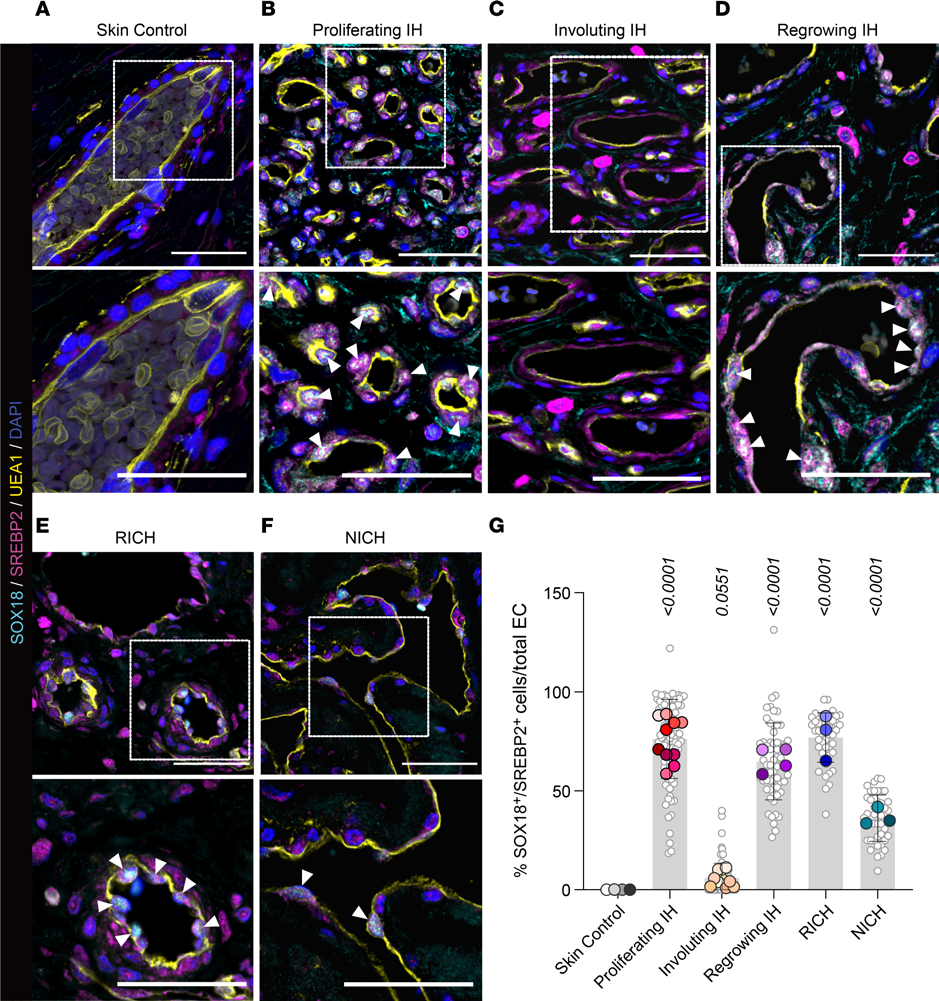
Nuclear colocalization of SOX18 and SREBP2 in IH and congenital vascular tumors. (**A**–**D**) Human age-matched skin, proliferating IH, involuting IH, and regrowing IH stained with anti-SREBP2 (magenta), anti-SOX18 (cyan), and the human endothelial cell–specific lectin UEA1 (yellow). Cell nuclei stained with DAPI (blue). (**E** and **F**) Rapidly involuting and non-involuting congenital hemangiomas (RICH and NICH) stained in the same manner. Boxed areas are shown enlarged in the bottom panels. (**G**) SOX18^+^SREBP2^+^ double-positive cell nuclei (arrowheads) were quantified and expressed relative to total endothelial cells. *P* values were calculated using 1-way ANOVA with Šidák correction. Data are shown as the mean ± SD. Data represent 15 representative images each for patient sample sizes *n* = 4 for age-matched skin controls, *n* = 10 each for proliferating- and involuting-phase IH, *n* = 4 for regrowing IH, and *n* = 3 each for RICH and NICH. See Table 1 for detailed patient information. Scale bars: 50 μm (top panels) and 25 μm (bottom magnified panels).

We investigated the nuclear localization of SOX18 and SREBP2 in histological sections from proliferating, involuting, and regrowing IH (Figure 4). We stained proliferating and involuting IH tissue sections (*n* = 10 each) from specimens excised from patients with IH who had not been treated with propranolol or corticosteroid (or any other treatment). Immunofluorescence staining of SOX18 and SREBP2 and staining with the lectin *Ulex europaeus* agglutinin I (UEA1), which specifically binds to human endothelial cells, showed colocalization of SOX18 and SREBP2 in endothelial nuclei throughout proliferating-phase IH sections (Figure 4, A–D, and Supplemental Figure 4.2). SOX18 was largely absent in involuting-phase IH. Age-matched human skin (*n* = 4), stained in parallel for comparison, was devoid of SOX18. SOX18 is not routinely detected in mature, quiescent blood vessels, as it is not needed to maintain an endothelial phenotype (27). We quantified SOX18^+^SREBP2^+^ nuclei/total endothelial nuclei and found a significant increase in proliferating but not in involuting IH when compared with normal skin (Figure 4G). Moreover, we analyzed tissue from 4 patients with regrowing IH (*n* = 4) (Figure 4D). These patients had received beta blocker treatment during infancy with notable clinical response. However, the IH regrew significantly after discontinuation of treatment. All regrowing IH specimens were positive for nuclear SOX18 and SREBP2 along the endothelium at levels comparable to those in proliferating IH and significantly increased in comparison with the skin control (Figure 4, A–D and G, and Supplemental Figure 4.2). All tissue stainings were validated using primary and secondary antibody controls (Supplemental Figure 4.2–4.4).

We further tested whether the SOX18-MVP axis is active in another pediatric vascular tumor, congenital hemangioma, with its sub-entities rapidly involuting and non-involuting congenital hemangioma (RICH and NICH) (28). We observed significantly increased SOX18^+^SREBP2^+^ endothelial cells in RICH and NICH compared with skin controls (Figure 4, E–G, and Supplemental Figure 4.2). This adds new insights into these understudied pediatric vascular tumors. In summary, the differential nuclear colocalization of SOX18 and SREBP2 in different stages of IH underscores the role of the MVP in IH pathogenesis and may serve as a marker to predict therapy response. In addition, we quantified Ki67^+^ proliferating cells in IH, RICH, and NICH and found significantly increased Ki67^+^ cells in RICH compared with normal skin controls, whereas proliferating, involuting, and regrowing IH and NICH were not significantly increased (Supplemental Figure 4.1). Table 1 provides detailed patient information for samples used in Figure 4.

### Statins inhibit vessel formation in a preclinical xenograft model of IH.

We surmised that if the MVP is critical to IH onset and progression, statins — competitive inhibitors of the rate-limiting enzyme in the MVP, HMGCR — would inhibit HemSC blood vessel formation in a preclinical IH model. The effect of statins on HemSCs has not been reported before, and therefore we first tested for cell toxicity. We chose to use atorvastatin, as it is among the most commonly used statins in cardiovascular disease patients, and simvastatin because of preexisting clinical trial data in infants with Smith-Lemli-Opitz syndrome (SLOS) (29). As atorvastatin is 5–10 times more potent than simvastatin, we adjusted the doses accordingly (30). To first address toxicity, HemSCs (*n* = 3 biological replicates) were treated with simvastatin (0.1–1 μM) or atorvastatin (0.01–0.1 μM). Neither statin affected HemSC viability at the given concentrations, compared with vehicle (DMSO) (Supplemental Figure 5.1).

**Figure 5:**
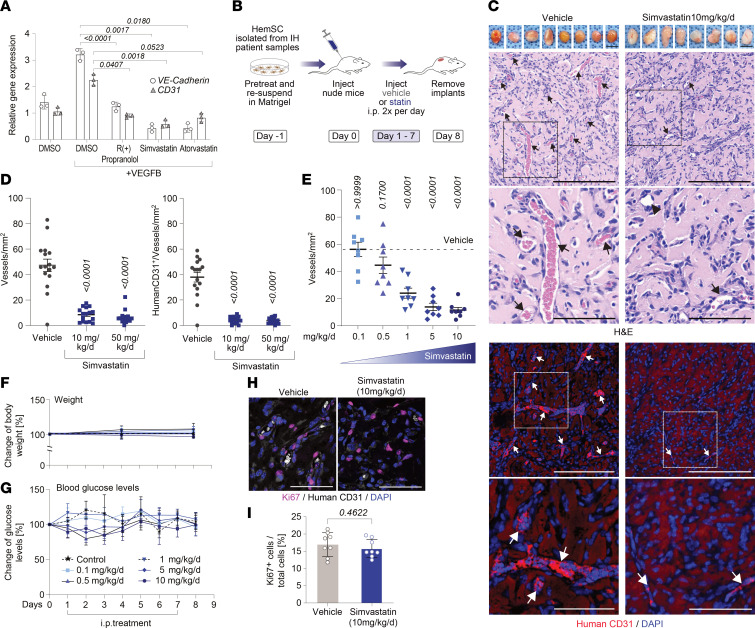
Statins inhibit HemSC endothelial differentiation and blood vessel formation. (**A**) HemSCs were induced to undergo endothelial differentiation for 6 days in the presence of simvastatin (0.5 μM), atorvastatin (0.1 μM), or R(+) propranolol (10 μM) (*n* = 3). VE-cadherin (circles) and CD31 (triangles) were measured by qPCR. R(+) propranolol served as positive control. (**B**) Schematic of IH xenograft model. (**C**) Top: Matrigel implants harvested after 7 days. Middle panels: H&E staining highlights blood vessel lumens with red blood cells. Bottom panels: Anti–human CD31 staining (red) indicates human blood vessels. Cell nuclei were stained with DAPI (blue). Arrows point at perfused, human CD31 positive vessels, arrowhead in the treatment group points at an immature vessel. Scale bars: 1 cm (Matrigel implants). Scale bars: 100 um (top); 50 um (bottom). (**D**) Left: Vessels/mm^2^ in H&E-stained sections. Right: Human CD31^+^ vessels/mm^2^. (**E**) Dose response to simvastatin. (**F** and **G**) Body weight (**F**) and blood glucose levels (**G**) of mice over 8 days. (**H** and **I**) Immunofluorescence staining for Ki67 and human CD31 in sections from control and simvastatin-treated mice (*n* = 8 biological replicates). Data were from 2 implants per mouse, leading to an observation sample size of *n* = 24 for vehicle (combined), *n* = 14 for 10 mg/kg/d and *n* = 16 for 50 mg/kg/d simvastatin (**D**), *n* = 10 for vehicle, and *n* = 10 for 10 mg/kg/d, *n* = 10 for 5 mg/kg/d, *n* = 10 for 1 mg/kg/d, *n* = 10 for 0.5 mg/kg/d, and *n* = 10 for 0.1 mg/kg/d simvastatin in the dose-response experiment in **E**. *P* values were calculated using 1-way ANOVA multiple-comparison test with Tukey’s correction (**A** and **D**), 1-way ANOVA multiple-comparison test with Dunnett’s correction (**E**), 2-way ANOVA multiple-comparison test with Dunnett’s correction (**F**), and a 2-tailed, unpaired *t* test (**I**). Data are shown as the mean ± SD.

Next, we tested 1 μM simvastatin and 0.1 μM atorvastatin on HemSC endothelial differentiation (12). Both statins significantly inhibited endothelial differentiation as indicated by decreased expression of the endothelial cell markers CD31 and VE-cadherin compared with vehicle control; R(+) propranolol was included as a positive control (Figure 5A). KLF2 and KLF4 were analyzed in the same HemSC to endothelial differentiation assay because of reported statin effects on these transcription factors (31, 32). Neither simvastatin, atorvastatin, nor R(+) propranolol increased KLF2 and KLF4 in differentiating HemSCs (Supplemental Figure 5.2).

We next tested whether statins would impact de novo vessel formation in the murine xenograft model using IH-derived HemSCs (*n* = 4) (Figure 5B). Simvastatin at 10 mg/kg/d (Figure 5, C and D) and atorvastatin at 1 mg/kg/d (Supplemental Figure 5.3) both significantly inhibited blood vessel formation seen by H&E staining and by anti–human CD31^+^ staining. A simvastatin dose-response experiment showed that 1 mg/kg/d was sufficient to significantly inhibit vessel formation (Figure 5E). Glucose levels and body weight of mice were unaffected (Figure 5, F and G). The human equivalent doses corresponding to the doses used in mice are shown in Supplemental Figure 5.12. The effective doses of simvastatin and atorvastatin in Figure 5 are below effective doses used in adults and significantly below the dose used in infants with SLOS (0.5–1 mg/kg/d simvastatin) (29, 33). Notably, the inhibitory effect of statins was limited to HemSC de novo vessel formation and did not impact angiogenic sprouting and ingrowth of surrounding murine vessels into the Matrigel implant (Supplemental Figure 5.4 and 5.5). In addition, Matrigel implant sections were stained and quantified for the proliferating cell maker Ki67. Ki67^+^ cells were comparable in both groups, indicating that proliferation per se was not affected by statins in this context (Figure 5, H and I), consistent with the lack of effect of statins on HemSC and HemEC proliferation in vitro (Supplemental Figure 5.6). Taken together, this supports why we observed reduced redness but no decrease in size of Matrigel implants in the statin-treated xenograft mice — reflecting that differentiation rather than proliferation is the key event in IH vessel formation (Figure 5C). Specificity of the anti–human CD31 and anti–mouse CD31 antibodies was verified (Supplemental Figure 5.7). In summary, our data show that simvastatin and atorvastatin inhibit endothelial differentiation of HemSCs in vitro and HemSC vasculogenesis in a preclinical IH xenograft model. This strongly suggests that the MVP contributes to vasculogenesis in IH and can be effectively targeted by statins in a translational approach.

We next tested statins in HemSC differentiating into microvascular mural cells (MMC) or adipocytes. MMC differentiation was induced by coculture with HemECs for 5 days followed by immuno-separation with anti-CD31 (13, 34); immune separation was confirmed (Supplemental Figure 5.8). The MMC genes *Calponin*, *PDGFRB*, *NG2*, and *TAGLN* were not affected by 0.1 μM atorvastatin or 0.5 μM simvastatin (Supplemental Figure 5.9). Similarly, atorvastatin or simvastatin treatment of HemSCs undergoing adipogenic differentiation had no effect over 8 days of differentiation, quantified by Oil Red O staining (Supplemental Figure 5.10). Rapamycin served as a positive control for inhibition of HemSC adipogenic differentiation (35). The adipogenic genes *PPARg*, *cEBPa*, and lipoprotein lipase (*LPL*) were similarly unaffected by statin treatment (Supplemental Figure 5.11). In summary, these data suggest that statins inhibit endothelial differentiation of HemSCs but not adipogenic or MMC differentiation.

### Statin, R(+) propranolol, and rapamycin upregulation of the low-density lipoprotein receptor in HemSCs.

**Figure 6:**
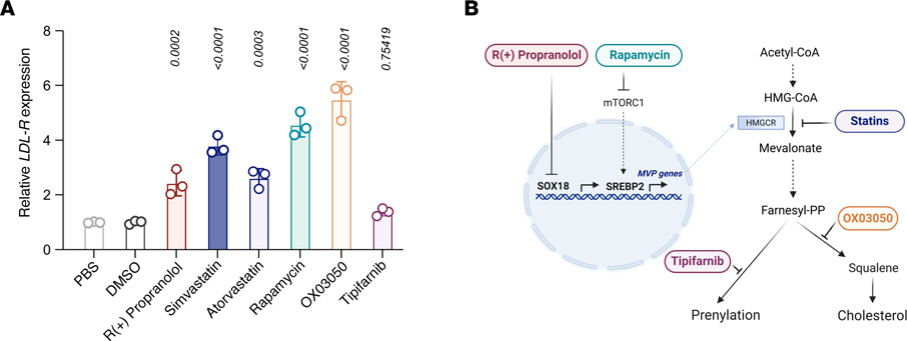
Drug modulation along the SOX18-MVP axis in HemSCs results in upregulation of *LDL receptor* mRNA levels. (**A**) *LDL receptor* mRNA was measured in HemSCs following 24-hour treatment with 10 μM R(+) propranolol; 0.5 μM simvastatin; 0.1 μM atorvastatin; 20 nM rapamycin; 28 nM OX03050, a squalene synthase 1 inhibitor; or 0.1 μM of the farnesyltransferase inhibitor tipifarnib. PBS [for R(+) propranolol] and DMSO (for all other drugs) served as vehicle controls. *P* values were calculated using 1-way ANOVA with Šidák correction. Data are shown as the mean ± SD (*n* = 2 biological replicates; 1 of the replicates was used in 2 independent experiments, resulting in 3 data points). (**B**) Schematic illustrates points of inhibition of the different drugs along the endothelial SOX18-MVP axis.

A well-documented effect of statins is increased low-density lipoprotein receptor (LDL-R) expression. This is a compensatory response to offset the HMGCR inhibition to maintain cellular cholesterol levels (36, 37). Thus, we analyzed *LDL-R* expression in HemSCs treated with R(+) propranolol or statins for 24 hours. Consistent with the R(+) propranolol inhibition of the MVP, R(+) propranolol significantly increased *LDL-R* expression to an extent similar to that seen with simvastatin and atorvastatin. We tested rapamycin as it has been used effectively for IH (38, 39) and because mTORC1 activates SREBP2 (40); hence rapamycin may also impact the MVP in HemSCs. Indeed, rapamycin significantly increased *LDL-R* levels. OX03050 — a squalene synthase 1 inhibitor — served as a positive control. Tipifarnib, a farnesyltransferase inhibitor acting downstream of the MVP, served as a negative control. Together, this provides another line of evidence for the response of HemSCs to statins and shows similar increases in *LDL-R* by R(+) propranolol, statins, and rapamycin. This suggests that combinations of these drugs targeting the SOX18-MVP axis at different levels might offer an innovative therapeutic approach to treat IH (Figure 6A). The schematic in Figure 6B illustrates points of inhibition for these drugs along the SOX18-MVP axis.

## Discussion

In this study, we uncover an endothelial SOX18-MVP axis as a central regulator of IH pathogenesis. The R(+) enantiomer of propranolol, shown previously to disrupt SOX18 transcriptional activities, downregulates expression of MVP genes in a SOX18-dependent manner, and it reduces mature SREBP2 protein, the master transcriptional regulator of the MVP. We propose that SOX18 augments the transcription of MVP genes to regulate cholesterol and isoprenoid biosynthesis in HemSCs undergoing endothelial differentiation. Competitive inhibition of the rate-limiting enzyme of the MVP — HGMCR — with statins results in significantly reduced HemSC blood vessel formation in a preclinical IH xenograft model. Together, these results make a compelling case for the involvement of an endothelial SOX18-MVP axis in the etiology of IH and suggest statins may be a new therapeutic strategy. In line with our previously discovered drug mechanisms in IH — including corticosteroids, sirolimus, and propranolol (12, 13, 35, 41) — statins inhibit HemSC endothelial differentiation and have little effect on proliferation.

Additional experiments support these insights. R(+) propranolol or the SOX18 inhibitor Sm4 decreased new synthesis of cholesterol in HUVECs, and the *RaOp* dominant-negative SOX18 (42) downregulated HMGCS1 and HMGCR. Moreover, SOX18 ChIP binding sites are present in *HMGCS1* and *HMGCR*. We next found increased SOX18 and SREBP2 colocalized in endothelial nuclei in proliferating-phase IH and 4 cases of regrowing IH, further connecting SOX18 to the MVP. Regrowing IH defies the classic IH life cycle, and little is known about how or why these IHs regrow (43); most often, regrowing IH involves oral mucosa, such as the lip. Typically, propranolol therapy is restarted to prevent sequelae. In our 4 patients with IH, regrowth occurred at 6–9 years of age. The nuclear colocalized SOX18 and SREBP2 in proliferating-phase IH and regrowing IH suggest that SOX18^+^SREBP2^+^ endothelial nuclei may serve as a biomarker for the vasculogenic capacity of the tumor.

SOX18 is a transcriptional regulator of vascular and lymphatic development, and tumor angiogenesis (16–18, 23, 44, 45). Notably, while glycolysis and fatty acid oxidation have been well established in physiological and pathological endothelial cell metabolism (46, 47), the MVP has not been implicated as a crucial regulatory pathway with the exception of one study in which single-cell RNA-Seq of angiogenic endothelial cells identified *SQLE*, the gene that encodes squalene monooxygenase, as a metabolic angiogenic target (48). Our discovery of a functional link between the endothelial cell–specific SOX18 and the MVP brings to light what we consider an entirely novel concept in endothelial differentiation and vasculogenesis.

The MVP is central to cholesterol biosynthesis and isoprenoid biosynthesis, the latter of which is needed for prenylation. The MVP bifurcates at farnesyl pyrophosphate (FPP) to produce squalene, an intermediate in cholesterol biosynthesis, or geranylgeranyl pyrophosphate (GGPP). FPP and GGPP are directly involved in prenylation of various small GTPases, and other protein substrates including RAS family members (49). Importantly, RAS signaling has been implicated in IH (50). The R(+) propranolol–mediated reduction in MVP genes and cholesterol biosynthesis may affect membrane fluidity and ruffling or activation of important signaling pathways via reduced prenylation. This warrants further investigation.

Pleiotropic benefits of statins, beyond lowering plasma LDL-cholesterol levels, are well documented. These include inhibiting inflammatory responses, increasing the bioavailability of nitric oxide, promoting re-endothelialization, and reducing oxidative stress (51–53). The underlying mechanisms, however, remain elusive. A recent study suggests epigenetic effects: simvastatin significantly improved human induced pluripotent stem cell–derived endothelial cell function by reducing chromatin accessibility under physiological and pathological conditions (54). Interestingly, mTORC1 reduces ER cholesterol levels, which in turn activates SREBP2 and the MVP (40), indicating that rapamycin may indirectly inhibit the MVP.

Statins are widely used drugs. The resulting reduction in cholesterol synthesis increases LDL receptor expression, which in turn clears LDL-cholesterol from the circulation. In our study, statins, R(+) propranolol, and rapamycin significantly increased LDL receptor transcript levels in HemSCs, suggesting convergence of these drugs, each with distinct mechanism, on the MVP. This suggests a potential for combination therapy with potentially lower doses of each drug and fewer adverse effects. Statins are generally considered a safe and well tolerated drug class. They can be associated with an increased risk of muscle pain, diabetes mellitus, and hepatic transaminase elevations; however, the risk of muscle symptoms from statin therapy is small and generally mild (55). We addressed potential side effects and did not observe a change in glucose levels or weight with simvastatin and atorvastatin in the xenografted mice over 7 days. A limitation is the relatively short treatment duration to detect adverse effects; moreover, adverse effects in rodents differ from those in humans. Bearing in mind drug safety as the highest priority when considering translating statins to infants, we performed a dose-response experiment with simvastatin. The lowest dose that showed significant reduction in IH vessels in the xenograft model was 1 mg/kg/d (Figure 5E). This translates to a human equivalent dose of 0.081 mg/kg/d (56) (Supplemental Figure 5.12), which is 6.25 times below the recommended dose of 0.5 mg/kg/d systemic simvastatin for infants with other indications. Further, topical rather than systemic statins might be used for superficial or less complex IH with increased safety as shown for other applications discussed below.

Systemic statins have been used safely in infants with Smith-Lemli-Opitz syndrome (SLOS), an autosomal-recessive syndrome characterized by the accumulation of 7-dehydrocholesterol. In a randomized, placebo-controlled clinical trial the recommended dose was 0.5 to 1 mg/kg/d (29, 33, 57, 58). Topical statins have been used for dermatological conditions such as the X-linked and dominant congenital hemidysplasia with ichthyosiform erythroderma and limb defects (CHILD) syndrome (59). In addition, statins have been applied to treat alopecia areata (60, 61). Based on the successful use of statins in children with these disorders, we suggest a safe repurposing of topical and/or systemic simvastatin to treat infants with IH.

It is exciting to speculate that the link between SOX18 and the MVP may have important implications for various pathophysiological conditions of the endothelium with disturbed vasculogenesis and angiogenesis, including developmental defects underlying vascular anomalies. Indeed, we found elevated SOX18^+^SREBP2^+^ endothelial cells in the congenital hemangioma entities RICH and NICH, suggesting that further exploration of the MVP in other vascular anomalies is warranted.

In contrast to vascular malformations for which genetic drivers have been identified, the genetic basis of IH is unknown. The known driver mutations in vascular malformations have facilitated precision medicine by repurposing existing oncology drugs (62, 63). In contrast, critical molecular players in IH have been uncovered by the study of mechanisms of action of serendipitously discovered drugs (12, 13, 35, 41). Herein, our discovery revealed a mechanistic link between the endothelial cell–specific transcription factor SOX18 and the MVP, enabling molecularly characterized, targeted treatment approaches for IH.

The endothelial SOX18-MVP axis functionally connects the mechanisms of action of beta blockers and statins. Of broader interest, beta blocker or statin use in oncology patients in addition to standard treatment has been shown to result in significantly improved cancer-related mortality in retrospective and prospective clinical trials in various entities (64–68). We speculate this may be due to the inhibition of the endothelial SOX18-MVP axis in tumor endothelial cells.

In summary, our findings uncover an endothelial SOX18-MVP axis as a central molecular driver for IH vasculogenesis. Based on SOX18 loss- and gain-of-function approaches, we surmise that SOX18 may act as an endothelial fine tuner for MVP activity. In a preclinical xenograft model with cells derived from patients with IH, we show that simvastatin or atorvastatin inhibits IH vessel formation. This suggests statins could be repurposed to treat this common vascular tumor of infancy, topically or systemically.

## Methods

### Sex as a biological variable

Our study used male nude mice to xenograft hemangioma-derived cells isolated from male and female patients with IH (see Table 1).

### IH cell isolation and culture

The clinical diagnosis of IH was confirmed in the Department of Pathology of Boston Children’s Hospital; IH specimens were deidentified as specified in Table 1. HemSCs and HemECs were selected from IH single-cell suspensions using anti-CD133– and anti-CD31–coated magnetic beads (Miltenyi Biotec), respectively, and expanded and cryopreserved. Cells were tested for mycoplasma by qPCR when cells were thawed and every 4–6 weeks thereafter. Cells were seeded on fibronectin-coated (0.1 μg/cm^2^; MilliporeSigma) plates at 20,000 cells/cm^2^ in Endothelial Growth Medium-2 (EGM-2; Lonza), which consists of Endothelial Basal Medium-2 (EBM-2; Lonza), SingleQuots (all except hydrocortisone and gentamicin-1000; Lonza), 10% heat-inactivated fetal bovine serum (FBS) (Cytiva), and 1× GPS (292 mg/mL glutamine, 100 U/mL penicillin, 100 mg/mL streptomycin; Mediatech). Hereafter, this full growth medium is referred to as EGM-2. Cells were cultured at 37°C in a humidified incubator with 5% CO_2_ and fed every other day.

### Hemangioma endothelial differentiation assay

HemSCs (samples denoted as HemSC 125, 149, 150, 165, 167, 171) were seeded on fibronectin-coated plates at a density of 20,000 cells/cm^2^ in EGM-2. After 18–24 hours, cells were washed with EBM-2 once and starved for 16 hours in 2% BSA/serum-free EBM-2. Cells were washed again once with EBM-2 and induced to undergo endothelial cell differentiation in serum-free EBM-2 containing 1× insulin-transferrin-selenium, 1:100 linoleic acid–albumin, 1 μM dexamethasone, and 100 μM ascorbic acid-2-phosphate with or without 10 ng/mL VEGF-B (R&D Systems) on day 0 and every 2 days thereafter. For addition of inhibitors, a preincubation with or without respective inhibitors for 30 minutes was conducted followed by continuous treatment. Stock solutions of 10 mM R(+) propranolol hydrochloride (MilliporeSigma), 105 mM simvastatin, and 10 mM atorvastatin (both MilliporeSigma) were prepared in DMSO (MilliporeSigma). Twenty micromolar R(+) propranolol was added for 2 hours and cells harvested for RNA-Seq (*n* = 6) (Figure 1, A–C). Continuous treatment with 1 μM simvastatin, 0.1 μM atorvastatin as well as 20 μM R(+) propranolol as a positive control was applied to differentiating HemSCs (*n* = 3) to test for an effect of statins in vitro (Figure 5A). Respective vehicle controls of each drug were used. Vehicle without VEGF-B served as a negative control and with VEGF-B as a positive control for differentiation.

### Hemangioma microvascular mural cell differentiation assay

HemSCs denoted as 171 (Table 1) were seeded together with endothelial colony-forming cells at a ratio of 1:1 at a total density of 30,000 cells/cm^2^ on fibronectin-coated plates in EGM-2, as previously described (69). DMSO (MilliporeSigma) was added as a negative control; 25 μM DAPT (MilliporeSigma) was added as a positive control. After 4 hours, the culture medium was changed to 0.5 μM simvastatin or 0.1 μM atorvastatin to determine the effect on microvascular mural cell (MMC) differentiation. The medium was changed every other day. Cells were cocultured for 5 days, harvested, and separated with immunomagnetic beads conjugated with anti-CD31 (Invitrogen) to obtain CD31^+^ (endothelial cells) and CD31^–^ (HemSCs and HemSC-derived MMCs) (see schematic in Supplemental Figure 5.8).

### Adipogenic differentiation assay

Racemic propranolol and R(+) propranolol (MilliporeSigma) were reconstituted at 10 mM in phosphate-buffered saline (PBS) for stock solution. Rapamycin (LC Laboratories) was reconstituted in DMSO to 10 mM for stock solution. On day –1, HemSCs were seeded at 20,000 cells/cm^2^ in fibronectin-coated wells (0.1 μg/cm^2^) on a 24-well plate and allowed to adhere overnight. Adipose mesenchymal stem cells served as controls. On day 0, cells were washed with PBS and incubated in adipogenic medium (AM), consisting of DMEM–high glucose (Gibco), 10% heat-inactivated FBS (Cytiva), 1× penicillin–streptomycin–l-glutamine (GPS; Corning), 1 μM dexamethasone (MilliporeSigma), 0.5 mM 3-isobutyl-1-methylxanthine (MilliporeSigma), 5 μg/mL insulin, and 60 μM indomethacin (both MilliporeSigma). AM without dexamethasone, IBMX, insulin, and indomethacin served as the control and was denoted as “control medium” in adipogenesis assays. The medium was replaced every 48 hours. Adipogenic differentiation of HemSCs was conducted over the course of 8 days, followed by Oil Red O staining and qPCR.

### Cholesterol measurements in HUVECs

HUVECs were grown in EBM-2 medium (Lonza, C2519A) supplemented with EGM-2 BulletKit (Lonza). HUVECs were seeded on 0.5% gelatin–coated 6-well plates at a density of 1.5 × 10^5^ cells per well overnight. The day after seeding, cells were treated with 2.5 mM MBCD (MilliporeSigma) to deplete cells of endogenous cholesterol. MBCD was withdrawn after 4 hours, and cells were washed with PBS and treated with either PBS, 20 μM R(+) propranolol (MilliporeSigma), DMSO, or 40 μM Sm4 (MilliporeSigma) for 18 hours. Cells were then washed with PBS, trypsinized, washed with PBS, and centrifuged. Lipids were extracted from the cell pellets through one-phase extraction with 202 μL of BuOH/MeOH (1:1) (MilliporeSigma) that contained 10 μM of cholesterol-d_7_ (Cayman Chemical) as the internal standard (70). The cholesterol analysis was performed on a TSQ Altis triple quadrupole mass spectrometer, operated in positive ion mode, coupled to a Vanquish UHPLC system (Thermo Fisher Scientific) using the transition from precursor mass of *m*/*z* 369.3516 (MS1) to *m*/*z* 161.1 (MS3) for cholesterol and 376.4 to 161.1 for cholesterol-d_7_ (70). The solvent pair included solvent A (100% H_2_O, 0.1% FA, and 2 mM NH_4_COO^–^) and solvent B (100% MeOH, 0.1% FA, and 2 mM NH_4_COO^–^) with a flow rate at 0.3 mL/min (80% B at the start). Cholesterol and its deuterated standard were separated on an Eclipse Plus C8 column (Agilent), and peaks were integrated with TraceFinder 5.1 (Thermo Fisher Scientific) using the daughter ion at *m*/*z* 161.1 (71) (Figure 2, B and C).

### RaOp expression in HUVECs

HUVECs (Lonza, C2519A) were seeded and transfected a day later using X-tremeGene HP Transfection Reagent kit (Roche) to introduce 500 ng of Halo-RaOp DNA using EBM-2 culture medium without antibiotic. Cells were incubated at 37°C with 5% CO_2_ overnight. The next day, cells were incubated with 5 nM of JF646-Halo ligand for 15 minutes, washed with PBS, and trypsinized; as a result, HUVECs expressing SOX18*^RaOp^* were fluorescently tagged. Cells were spun down, washed with PBS, and permeabilized with 0.2% Triton X-100 for 10 minutes, followed by blocking with 5% BSA/PBS for 1 hour. Cells were labeled with 1:300 dilution of anti–human HMGCR (Thermo Fisher Scientific, PA537367) or HMGCS1 (Thermo Fisher Scientific, PA513604) for 1 hour at room temperature in the dark. Cells were washed with PBS, stained with 1:500 dilution of anti-rabbit IgG conjugated with Alexa Fluor 488 (Invitrogen, A11008) for 30 minutes, and washed again with PBS. Cells were then imaged on a Fortessa X-20 (BD Biosciences). Data analysis was performed using FlowJo software.

### Lentiviral overexpression and knockdown of SOX18

Undifferentiated HemSCs were transduced with a lentivirus encoding SOX18 linked to GFP (custom lentivirus VB240403-1251jyg; Vector Builder) or an empty vector control virus (control lentivirus VB010000-9298rtf; Vector Builder). Successful lentiviral transduction was confirmed by nuclear GFP expression, and overexpression efficiency was confirmed by WB (Supplemental Figure 3.3). Lentiviral knockdown of SOX18 in HemECs was performed with SOX18 shRNA lentivirus (TRCN0000017450, MilliporeSigma) or an empty vector control virus (SHC001V, MilliporeSigma) followed by puromycin (1 μg/mL) selection for 5 days. Thereafter, cells were maintained in EGM-2. Knockdown efficiency was confirmed by qPCR and WB. R(+) propranolol treatment of HemSC^SOX18OE^ and HemEC^shSOX18^ (samples denoted as HemSC^SOX18OE^ 171 and HemEC^shSOX18^ 133, 150, and 171) was for 24 hours.

### Western blot

HemSCs were induced to undergo differentiation according to the protocol described above. Cells were treated with 20 μM R(+) propranolol for 2 hours on day 6 of endothelial differentiation and lysed in a RIPA-based lysis buffer (25 mM Tris-HCl [pH 7.6], 150 mM NaCl, 1% NP-40, 1% sodium deoxycholate, 0.1% SDS) with protease and phosphatase inhibitors (Cell Signaling Technology) as well as calpain I and proteasome V inhibitors (MilliporeSigma). Cell extracts were electrophoresed by SDS-PAGE, transferred to nitrocellulose or to PDVF, and probed with anti-SREBP2 clone 22D5 (72) (1:200; MilliporeSigma, MABS1988) followed by anti-GAPDH (1:2,000; Cell Signaling Technology, 5014). For protein quantification of SOX18 overexpression efficiency in HemSC^SOX18OE^ and knockdown efficiency in HemEC^shSOX18^, cells were seeded on fibronectin-coated plates (0.1 μg fibronectin/cm^2^) at a density of 20,000 cells/cm^2^ in EGM-2 medium, lysed, and processed as described above after staining with SOX18 (D-8) (1:500; Santa Cruz Biotechnology, sc-166025) and GAPDH (Cell Signaling Technology) antibodies. All signals were detected by ECL. The densitometric analysis was conducted using Fiji ImageJ software.

### In vivo murine model for human blood vessel formation

Experiments were carried out with 3 × 10^6^ HemSCs per implant. HemSCs (*n* = 4, samples 125, 147, 149, 150) were grown in EGM-2 medium until 90% confluent. A stock solution of simvastatin (105 mM; MilliporeSigma) or atorvastatin (10 mM; MilliporeSigma) was prepared in DMSO. Twenty-four hours before trypsin removal from plates, 1 μM simvastatin or 0.1 μM atorvastatin or the equivalent DMSO concentration as a control was added to the medium. Cells were counted after the 24-hour pretreatment and suspended in 200 μL Matrigel (Corning) including 1 μg/mL basic FGF (ProSpec), 1 μg/mL erythropoietin (ProSpec), and 0.5 μM (simvastatin) or 0.05 μM (atorvastatin) or vehicle (DMSO) on ice. The Matrigel/cell suspensions were injected subcutaneously into the flanks of 6- to 7-week-old male athymic nude mice, strain Hsd: Athymic Nude-Foxn1nu (Envigo, 6904M), placing 2 implants per mouse (*n* = 3–5 mice per group; Figure 5B). The mice were treated with 0.1–50 mg/kg/d simvastatin, 1–15 mg/kd/d atorvastatin, or the equivalent DMSO concentration as a control (200 μL/mouse, i.p.) every 12 hours. Blood glucose levels were measured daily before the morning i.p. injection. Glucose concentrations were measured in tail vein blood using the OneTouch UltraSmart Blood Glucose Monitoring System (LifeScan). Body weight was measured before the injections (day 0), on day 4, and before removal of the implants (day 8). After 8 days, the mice were euthanized, and the implants were removed, photographed, fixed in formalin, embedded in paraffin, and analyzed by H&E staining and immunofluorescence staining. Blood vessels (indicated by luminal structures containing one or more RBCs) and CD31^+^ stained human vessels were counted in 5 fields per section, 2 sections per implant. Each field was 425.1 μm × 425.1 μm = 0.18071 mm^2^, and sections were from the middle of the implant. Vessel density is expressed as vessels per square millimeter.

### H&E and immunofluorescence staining of Matrigel implant and human FFPE tissue sections

FFPE tissue sections (5 μm) of the Matrigel implants were deparaffinized and either directly stained with H&E or immersed in an antigen retrieval solution (citrate-EDTA buffer containing 10 mM citric acid, 2 mM EDTA, and 0.05% Tween-20, pH 6.2) for 20 minutes at 95°C–99°C. Sections were subsequently blocked for 30 minutes in TNB Blocking Buffer (PerkinElmer) followed by incubation with a mouse anti–human CD31 monoclonal antibody (1:30; Dako, 0823) to stain for human endothelium. Next, sections were incubated with Alexa Fluor 647 chicken anti-mouse IgG (1:200; Invitrogen, A-21463) as a secondary antibody. An anti-mouse-specific CD31 monoclonal antibody (1:100; R&D Systems, AF3628) was used to quantify mouse vessels in the Matrigel implants. Alexa Fluor 647 chicken anti-goat IgG was applied as a secondary antibody (1:200; Invitrogen, A-21469). Tissue specificity of the anti-human and anti-mouse antibodies was confirmed by negative staining in mouse lung and human skin tissue, respectively (see Supplemental Figure 5.7). FFPE tissue sections (5 μm) from patients with IH or with congenital hemangioma were deparaffinized, immersed in an antigen retrieval solution, and blocked for 30 minutes in 10% donkey serum followed by incubation with mouse anti–human SOX18 (D-8) (1:50; Santa Cruz Biotechnology, sc-166025), rabbit anti–human SREBP2 (1:100; Abcam, ab30682), or rabbit anti–human Ki67 (1:100; Abcam, ab15580) and UEA1 fluorescently labeled with Alexa Fluor 649 (1:50; Vector Laboratories, DL1068). Next, the sections were incubated with Alexa Fluor 488 donkey anti-mouse IgG (1:200; Invitrogen, A-21202) and Alexa Fluor 546 donkey anti-rabbit IgG (1:200; Invitrogen, A-10040) as secondary antibodies. All slides were mounted using DAPI (Molecular Probes) to visualize nuclei. Immunofluorescence images were acquired by an LSM 880 confocal microscope (Zeiss). Images were analyzed through a ×20 or ×63 objective lens. All images were analyzed using Fiji ImageJ software.

### Oil Red O staining

The Oil Red O working solution (ORO; MilliporeSigma) was freshly prepared in a 3:2 ratio of 0.5% (wt/vol) ORO in isopropanol (Fisher Scientific) and double-distilled H_2_O (ddH_2_O) and was filtered to remove precipitates. For ORO staining of lipid droplets, 12 mm coverslips (Electron Microscopy Sciences) were coated with fibronectin (0.1 μg/cm^2^) in 24-well plates before seeding of HemSCs. Cells were fixed in 4% paraformaldehyde (Electron Microscopy Sciences) for 15 minutes at room temperature, washed in PBS, and incubated in 60% isopropanol for 3 minutes. Cells were stained with ORO for 10 minutes at room temperature and washed with 60% isopropanol for 10 minutes to remove unbound ORO dye. Cells were washed in ddH_2_O, mounted onto slides, and imaged within 24–48 hours to avoid precipitation.

### Immunofluorescence cell staining

HemEC^Ctr^ and HemEC^shSOX18^ were seeded on fibronectin-coated plates at a density of 30,000 cells/cm^2^ in EGM-2 medium for 30 hours on 2 cm^2^ slides, fixed in 4% paraformaldehyde, and blocked in 5% BSA/0.3% Triton X-100 for 1 hour. The mouse anti–human SOX18 used for immunostaining (D-8) was validated in HemEC^Ctr^ and HemEC^shSOX18^ (1:100; Santa Cruz Biotechnology, sc-166025) (Figure 3, L and M). Cells were costained with a rabbit anti–human SREBP2 antibody (1:200; Abcam, ab30682). Secondary antibodies included Alexa Fluor 488 donkey anti-mouse IgG (1:200; Invitrogen, A-21202) and Alexa Fluor 546 donkey anti-rabbit IgG (1:200; Invitrogen, A-10040). DAPI (Molecular Probes) was used to visualize nuclei, followed by mounting (Invitrogen). Immunostainings of IH sections with respective isotype-matched control IgG and secondary antibodies were conducted.

### RNA isolation and qPCR

Total RNA was extracted from cells with the RNeasy Micro Extraction Kit (QIAGEN). Reverse transcriptase reactions were performed using an iScript cDNA Synthesis Kit (Bio-Rad). qPCR was performed using SYBR FAST ABI Prism 2× qPCR Master Mix (Kapa BioSystems). Amplification was carried out in a QuantStudio 6 Flex Real-Time PCR System (Fisher Scientific). A relative standard curve for each gene amplification was generated to determine the amplification efficiency, with greater than 90% considered acceptable. Fold increases in gene expression were calculated according to the ΔΔCt method, with each amplification reaction performed in duplicate or triplicate (73). Gene expression was normalized to the PBS or DMSO treatment, respectively. ATP5B was used as housekeeping gene expression reference. A list of all primer sequences used in this study is given in Table 2.

### Proliferation assay

Cells were plated on 96-well plates with 1,500 cells per well and cultured in EGM-2 for 4 hours. The medium was removed and replaced with 0.1 mL of fresh EGM-2 containing R(+) propranolol, simvastatin, atorvastatin, OX03050, or rapamycin (all MilliporeSigma). The plates were incubated for 24 and 48 hours. After the treatment, 20 μL of MTS (CellTiter 96 AQueous One Solution Cell Proliferation Assay, Fisher Scientific) labeling reagent was added to each well, and plates were incubated for another 2 hours. The spectrophotometric absorbance of the samples was detected using a FilterMax F3 microplate reader (Molecular Devices) at 492 nm.

### Bioinformatics analysis

#### Bulk RNA-Seq.

We used Trimmomatic v0.39 (74) to trim the low-quality next-generation sequencing reads (-threads 20 ILLUMINACLIP:TruSeq3-PE.fa:2:30:10 LEADING:3 TRAILING:3 SLIDINGWINDOW:4:15 MINLEN:36). Subsequently, only the high-quality trimmed reads were aligned to the human reference genome (hg38) using STAR v2.7.2b (75). The read counts were calculated by featureCounts software (76). Differentially expressed genes were identified using the DESeq2 R package (adjusted *P* value < 0.05). KEGG pathways and Gene Ontology (GO) enrichment tests were performed by the clusterProfiler R package. A pathway or GO term was treated as significantly enriched if an adjusted *P* value (with Benjamini-Hochberg correction) was smaller than 0.05. The bar plots illustrating significant pathways or GO terms were created using the enrichplot R package.

#### ChIP-Seq.

The data are displayed on the UCSC genome browser. The layered epigenetic marks dataset was supplied from Encode. The ChIP-Seq binding location dataset can be found at https://www.ebi.ac.uk/biostudies/arrayexpress/studies/E-MTAB-4481

### Statistics

Data were analyzed and plotted using GraphPad Prism 10.1.0 (GraphPad Software). Results are displayed as the mean ± SD. For experiments in which cells were treated with drugs, the differences were assessed by 1-way ANOVA. Tukey’s post hoc test was used for multiple comparisons of different treatment modalities and Šidák’s, Tukey’s, or Dunnett’s test for multiple comparisons to compare every treatment mean with that of the respective vehicle control. A 2-tailed, unpaired *t* test was applied for comparisons between treatment and control groups given equal variance. Differences were considered significant for *P* values less than 0.05. Figures were created using Illustrator (Adobe Inc.); schematics were in part created using BioRender.com.

### Study approval

Animal protocols complied with NIH Animal Research Advisory Committee guidelines and were approved by the Boston Children’s Hospital IACUC (protocol 00001741). IH specimens were obtained under a protocol approved by Boston Children’s Hospital (IRB protocol 04-12-175R; AH, HK, JBM, JB) as well as Stanford University (IRB protocol 35473; RL, JT). Hemangioma specimens were collected upon written informed consent of the patient’s guardian, deidentified, and used for cell isolation under IRB-approved protocol 04-12-175R and in accordance with Declaration of Helsinki principles.

### Data availability

The bulk RNA-Seq data can be accessed at the Gene Expression Omnibus archive at the National Center for Biotechnology Information under accession number GSE274946 (day 6) and GSE275019 (day 4). The data are displayed on the UCSC genome browser. The layered epigenetic marks dataset was supplied from Encode. The ChIP-Seq binding location dataset can be found at https://www.ebi.ac.uk/biostudies/arrayexpress/studies/E-MTAB-4481 The supporting data values are compiled in a supplemental XLS file.

## Author contributions

AH, MSG, MF, and JB designed the study. AH, MSG, JWS, JWHT, MAH, LB, and LC conducted experiments. AH, MSG, JWS, JWHT, MAH, LB, SN, LC, AJ, MS, and PB carried out formal analysis. AH, LS, YQ, MV, MF, and JB were involved in supervision. RL, JT, HK, and JBM provided patient specimens and clinical expertise. AH performed patient data curation. AH and JB wrote the original manuscript. All authors reviewed, edited, and agreed to the final version of the manuscript. AH, MV, MF, and JB acquired funding.

## Supplementary Material

Supplemental data

Unedited blot and gel images

Supporting data values

## Figures and Tables

**Table 1:**
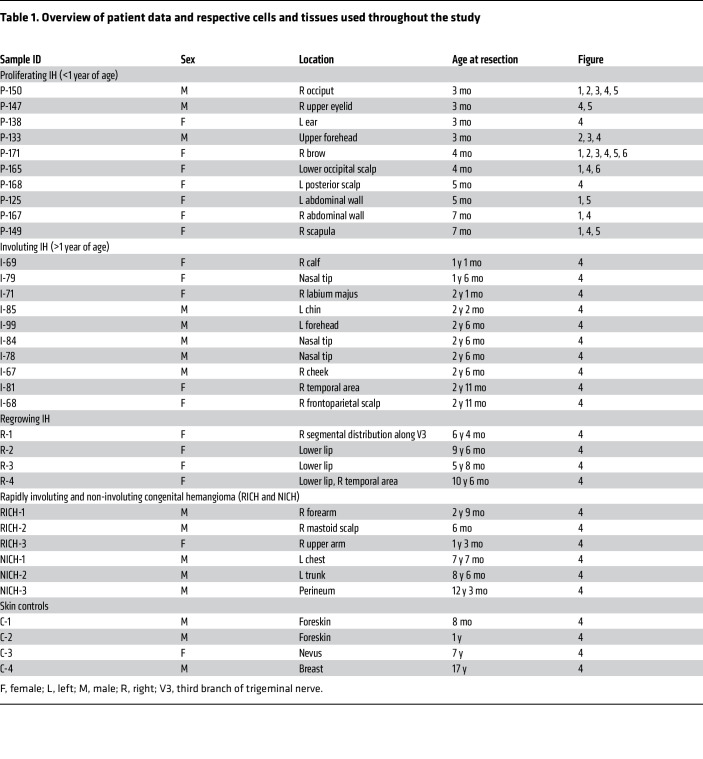
Overview of patient data and respective cells and tissues used throughout the study

**Table 2:**
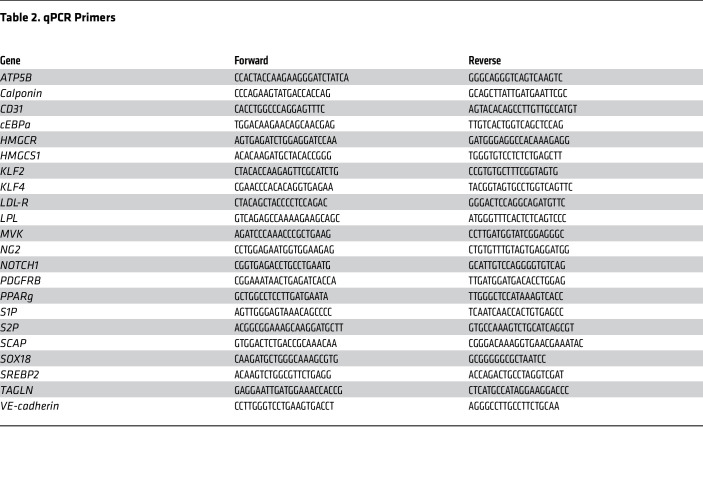
qPCR Primers

## Version 1 02/25/2025

In-Press Preview

## Version 2 04/01/2025

Electronic publication
