# Supplementary material for: An endothelial SOX18–mevalonate pathway axis enables repurposing of statins for infantile hemangioma
Source: J Clin Invest. 2025 Feb 25;135(7):e179782. doi: 10.1172/JCI179782 (PMC11957709; doi:10.1172/JCI179782)
Supplement: Supplemental data [file jci-135-179782-s268.pdf]

An endothelial SOX18-mevalonate pathway axis enables repurposing of statins for infantile hemangioma

Annegret Holm[1], Matthew S. Graus[2], Jill Wylie-Sears[1], Jerry Wei Heng Tan[1], Maya Alvarez-Harmon[1], Luke Borgelt[1], Sana Nasim[1], Long Chung[2], Ashish Jain[3], Mingwei Sun[3], Liang Sun[3], Pascal Brouillard[4], Ramrada Lekwuttikarn[5], Yanfei Qi[2], Joyce Teng[5], Miikka Vikkula[4,6], Harry Kozakewich[7], John B. Mulliken[8], Mathias Francois[2,9], and Joyce Bischoff[1*]

Supplemental Figures and Tables

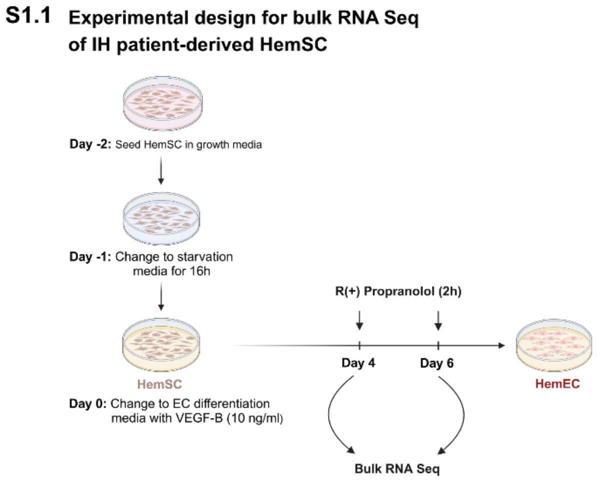

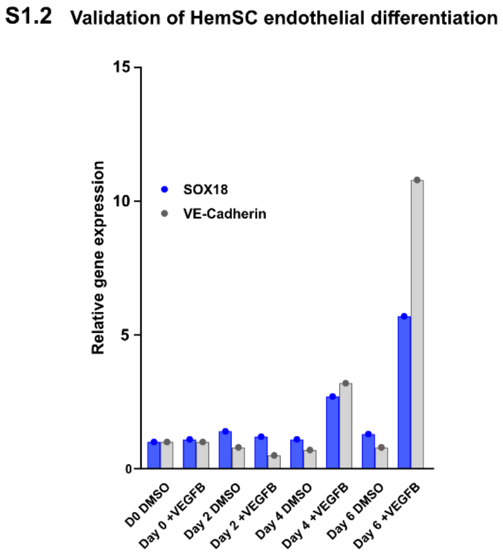

Supplemental Figure 1.

S1.1 Experimental steps to induce HemSC to undergo endothelial differentiation (n=6 biological replicates). VEGF-B at 10ng/ml was added to serum starved HemSC on Day 0. RNA isolated from cells treated ± R(+) propranolol (20 µM) for 2 hours on Day 4 and Day 6.

S1.2 HemSC to endothelial differentiation over 6 days was assessed by qPCR for SOX18 and VE-Cadherin (n=1).

Supplemental Figure 2. Overview of MVP genes regulated by R(+) Propranolol (Figure 1A-C)

	Day 4		Day 6	
Gene	*Log₂ Fc*	*Adjusted p value*	*Log₂ Fc*	*Adjusted p value*
HMGCS1	0.462979006	0.026354098	-1.63371667	0.000484519
HMGCR	0.169344377	0.044323597	-1.374323007	0.000424613
MVK	0.314895512	0.029933298	-1.147210743	0.000127633
MVD	0.441665738	1.75E-06	-1.288125115	0.002855957
FDPS	0.217777666	0.019564343	-1.033766375	0.006204092
IDI1	0.24599104	0.010011861	-1.226584676	0.004495371
FDFT1	0.173687183	0.080502823	-0.874992512	0.011617406
SQLE	0.186643474	0.080502823	-1.274290854	0.001552676
LSS	0.161258298	0.088436956	-0.74862184	0.017189697
SC5D	0.079030565	0.728721351	-0.96705494	0.005332315
HSD17B7	0.211229037	0.550897945	-1.81198247	2.01E-06
NSDHL	0.192680874	0.220906602	-0.686486501	0.015844376
DHCR7	0.231028296	0.09552771	-1.404321837	0.002936361
DHCR24	0.039092468	0.885153473	-1.599436532	0.014079728
ABCA1	-0.068070967	0.859222104	2.207383944	6.43E-05

Supplemental Figure 2. Log2 fold changes and adjusted p values of differentially regulated MVP genes and ABCA1 upon R(+) propranolol treatment on Day 4 and Day 6 shown in Figure 1B.

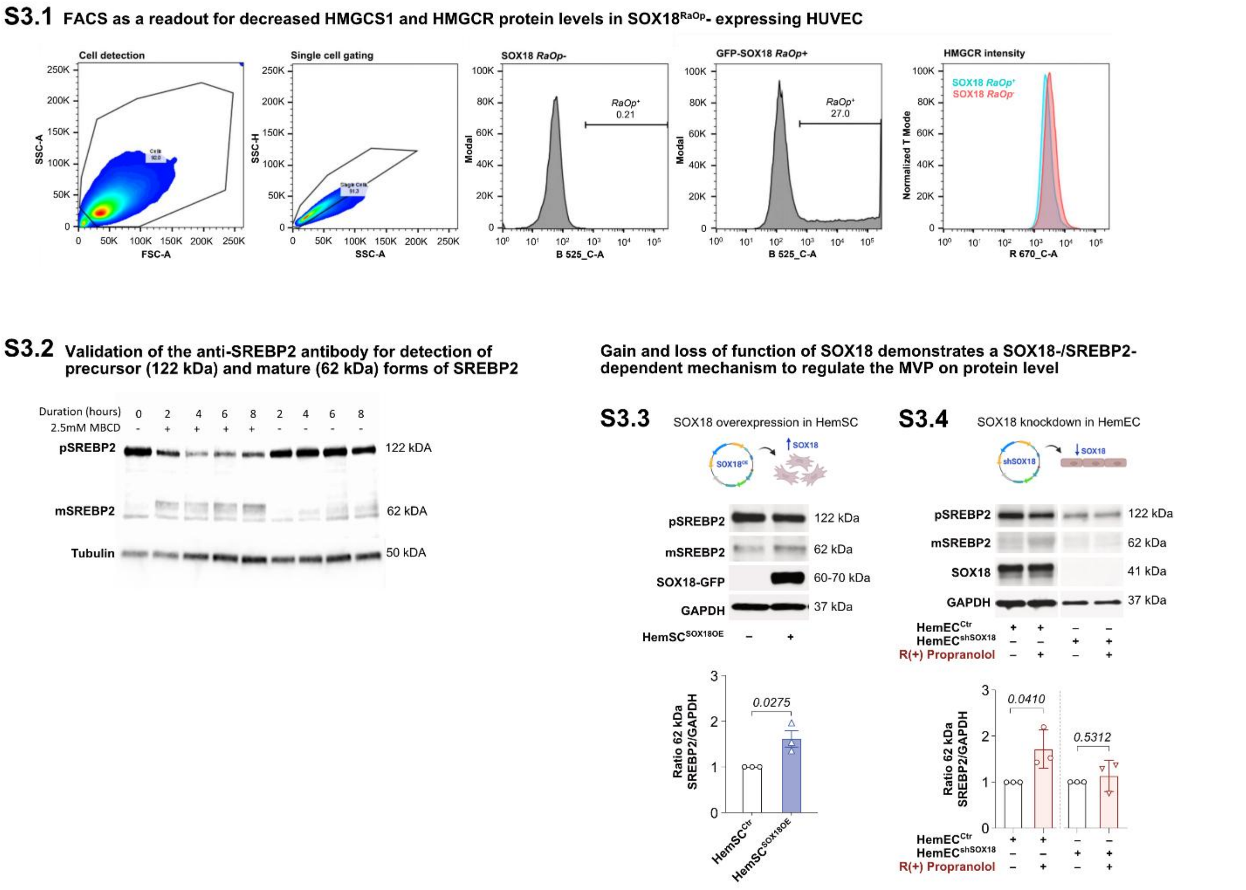

Supplemental Figure 3.

S3.1 Flow cytometry plots of HUVECs expressing fluorescently tagged SOX18^{RaOp} stained for HMGCS1 and HMGCR and respective control for analysis in Figure **3C**,**D**. Gating is shown in the two left panels; representative GFP expression in *RaOP*$^{+}$ and flow cytometry plot depicting decreased HMGCR intensity in *RaOP*$^{+}$ compared to its control in the two right panels.

S3.2 The human anti-SREBP2 antibody was validated in HemSC ± cholesterol depletion with MBCD for time points ranging from 0-8 hours. MBCD treatment corresponds with a transient decrease in 122 kDa precursor SREBP2 and corresponding increase in 62kDa mature SREBP2.

S3.3 WB analysis of HemSC with lentiviral overexpression of SOX18 (HemSC^{SOX18OE}), grown in full media (10% FBS), showed significantly increased mature SREBP2 (n=3 independent experiments). SOX18 overexpression verified by WB.

S3.4 WB analysis of control HemEC (HemEC^{Ctr}) versus HemEC with SOX18 knockdown (HemEC ^{shSOX18}) grown in full media (10% FBS) treated ± R(+) propranolol for 24 hours (n=3 biological replicates). SOX18 knockdown verified by WB.

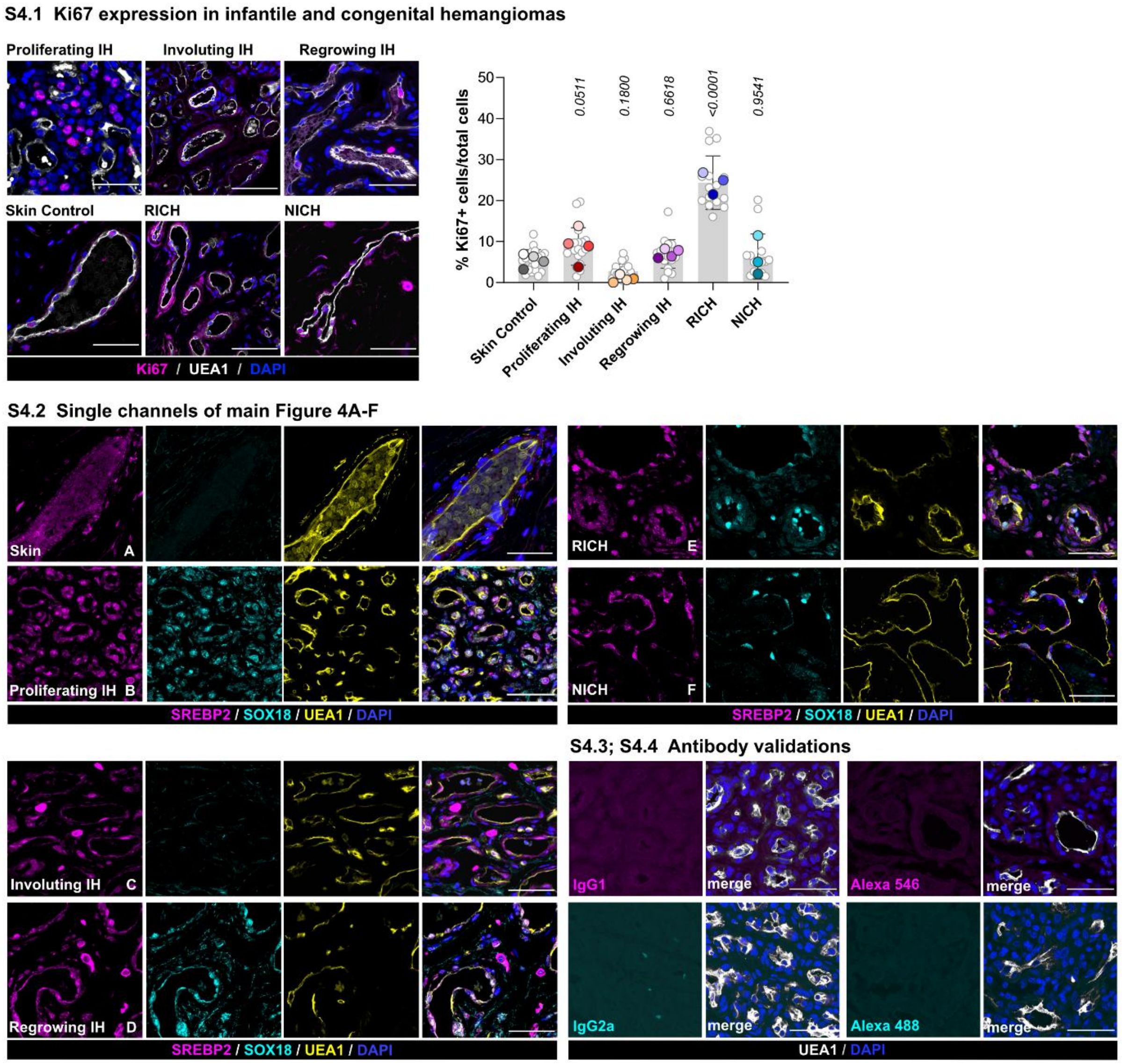

Supplemental Figure 4:

S4.1 Proliferation was assessed by staining with anti-Ki67 (magenta) in IH, RICH and NICH with skin as a control; vessels were stained with human EC-specific lectin UEA1 (grey), cell nuclei were stained with DAPI (blue). Quantification of Ki67 positive cells/total cells with ImageJ shows Ki67 significantly increased in RICH compared to normal skin (n=4 biological replicates for proliferating, involuting, regrowing IH, and skin control; n= 3 for RICH and n=3 NICH; each colored data point shows the average of 5 representative images each represented as a gray datapoint. P values were calculated using one-way ANOVA with Šidák-correction. Data show the mean ± SD; scale bars 50 µm.

S4.2 Single fluorescent channels of merged images in main Figure 4A-F for each antibody including SREBP2 (magenta), SOX18 (cyan), and the human specific lectin UEA1 (yellow) representing skin control, proliferating IH, involuting IH, regrowing IH, RICH, and NICH.

Validation of isotype-matched (**S4.3**) and secondary antibodies (**S4.4**) used in the study (proliferating IH tissue); scale bars 50 µm.

S5.1 Statins do not affect viability of HemSC

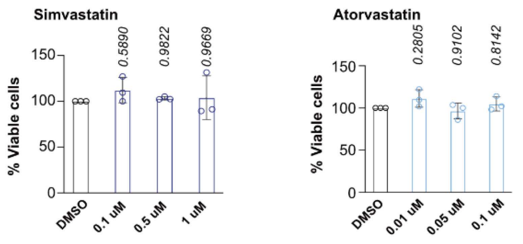

S5.2 Statins do not increase *KLF2* and *KLF4* in HemSC undergoing endothelial differentiation

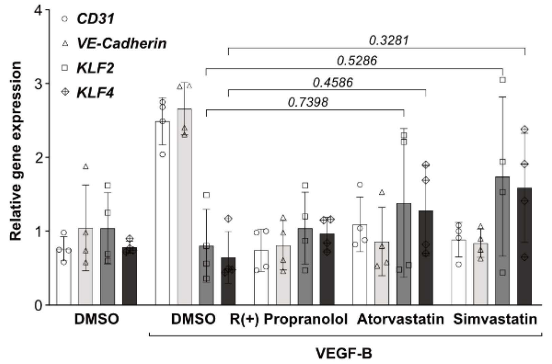

S5.3 Atorvastatin inhibits HemSC vessel formation in vivo

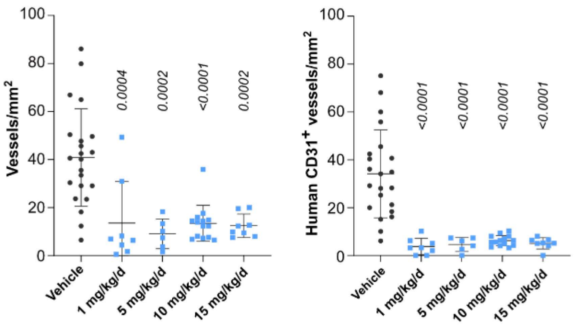

S5.4 Statins do not affect murine angiogenesis

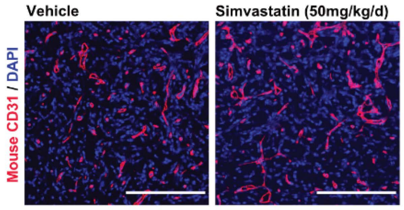

S5.5

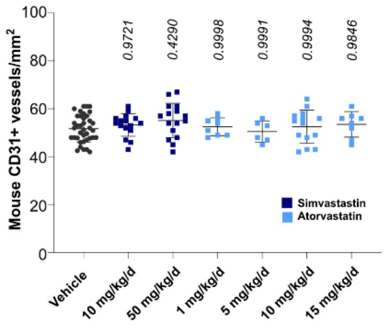

S5.6 Statins do not affect proliferation of HemEC and HemSC

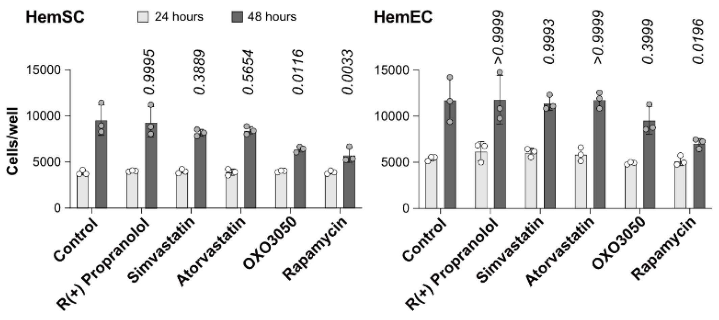

S5.7 Human and mouse CD31 antibody specificity

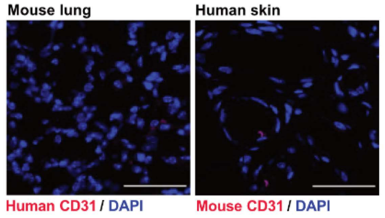

S5.8 Immunoseparation of CD31+/CD31- cells

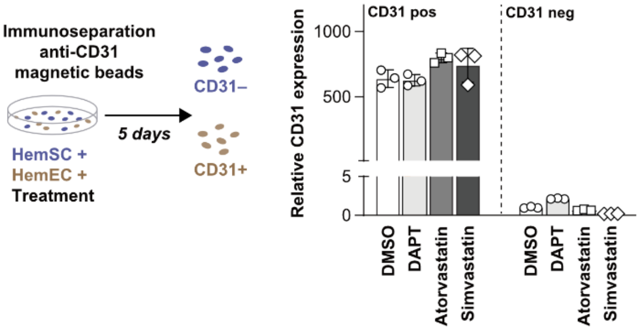

S5.10 Statins do not affect adipogenic differentiation of HemSC

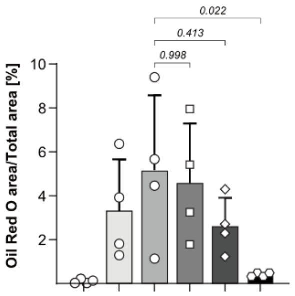

S5.11

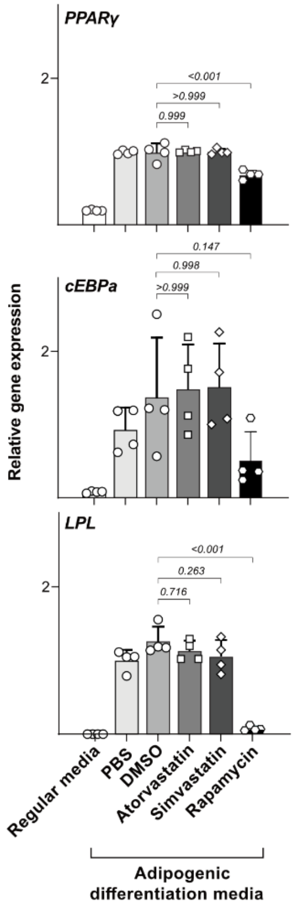

S5.9 Microvascular mural cell marker expression is not affected by statins

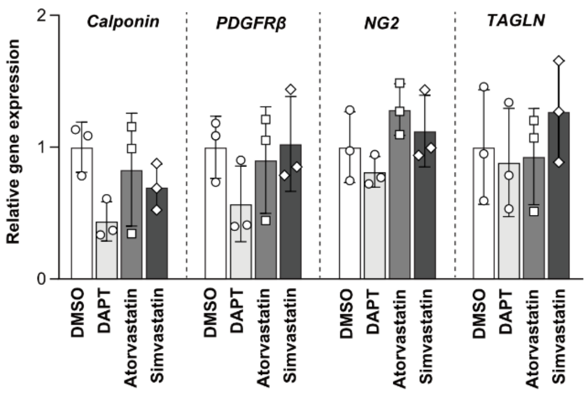

Table S5.12: Conversion of mouse to human statin doses

**Simvastatin**						
Used in mice (mg/kg/d)	**50**	**10**	**5**	**1***	**0.5**	**0.1**
Human equivalent dose (mg/kg/d)	4.065	0.813	0.407	0.081	0.041	0.008
**Atorvastatin**						
Used in mice (mg/kg/d)	**15**	**10**	**5**	**1***		
Human equivalent dose (mg/kg/d)	1.220	0.813	0.407	0.081		

Supplemental Figure 5.

S5.1 Simvastatin (0.1 - 1 µM) or atorvastatin (0.01 - 0.1 µM) had no effect on cell viability in HemSC treated for 48 hours).

S5.2 KLF2 and 4 mRNA levels measured by qPCR in HemSC undergoing endothelial differentiation in the presence of R(+) propranolol, atorvastatin, or simvastatin were unchanged on Day 6 compared to DMSO.

S5.3 HemSC (n=4) were pretreated with 0.1 μM atorvastatin or vehicle (DMSO) for 24 hours, suspended in Matrigel with 0.05 μM atorvastatin or an equivalent DMSO concentration and injected subcutaneously into nude mice with 2 implants/mouse. Mice were treated with 1, 5, 10 or 15 mg/kg/d atorvastatin or an equivalent volume of PBS with a DMSO every 12 hours for 7 days. Treatment with atorvastatin resulted in a significant reduction in vessel formation at each dose. Vessel density is expressed in vessels/mm^2.

S5.4 and S5.5 Matrigel implant sections from vehicle and statin treated mice were stained with anti-mouse CD31 and DAPI. The density of murine CD31+ blood vessels in the Matrigel implants was unaffected by either simvastatin or atorvastatin compared to vehicle (quantified in **S5.5**).

S5.6 Proliferation of HemSC and HemEC measured at 24 and 48 hours was not significantly reduced upon treatment with R(+) propranolol (10 μM), simvastatin (0.5 μM), or atorvastatin (0.1 μM). The squalene synthase 1 inhibitor OX3050 (28 nM) and rapamycin (20 nM) served as positive controls.

S5.7 Staining of murine lung with the anti-human CD31 used in Figure 5 and human skin with the anti-mouse CD31 used in S5.4 demonstrate antibody specificity for human or mouse CD31, respectively.

S5.4, **S5.7** Scale bars 100 μm.

P values were calculated using one-way ANOVA multiple comparisons test with Dunnett-correction **(S5.1)**, one-way ANOVA with Šidák-correction **(S5.2)**, one-way ANOVA multiple comparisons test with Tukey-correction (**S5.3, S5.4**). Data show the mean ± SD and were collected for 2 implants in each mouse, leading to an observation sample size of n=22 for vehicle (combined), n=8 (1mg/kg/d), n=6 (5 mg/kg/d), n=14 (10 mg/kg/d), and n=8 (15 mg/kg/d).

Statins had no effect on microvascular mural cell (MMC) (**S5.8**,**9**) and adipogenic (**S5.10**,**11**) differentiation of HemSC as demonstrated by mRNA levels of MMC genes *Calponin*, *PDGFR-B*, *NG2*, and *TAGLN*. Differentiating cells were treated with 0.1 μM Atorvastatin or 0.5 μM Simvastatin (n=3 biological replicates). mRNA levels of adipogenic transcription factors *PPARg* and *cEBPa* as well as *LPL* were measured upon treatment with 0.1 μM Atorvastatin, 0.5 μM Simvastatin, or 20 nM Rapamycin over the course of an 8-day adipogenic differentiation protocol. Rapamycin served as a positive control. Oil-Red-O staining quantified per total vessel area [%] confirmed statins did not affect adipogenic differentiation (n=4 biological replicates). P values were calculated using one-way ANOVA multiple comparisons test with Šidák-correction; Data show the mean ± SD.

S5.12 compares the reduced vessel density observed at 1 mg/kg/d for simvastatin and atorvastatin (*) to the calculated human equivalent doses of simvastatin and atorvastatin(1). The red box highlights the human equivalent dose of simvastatin used in infants with Smith-Lemli-Opitz syndrome (0.5-1 mg/kg/d).

Reference:

1. Nair AB, and Jacob S. A simple practice guide for dose conversion between animals and human. *J Basic Clin Pharm.* 2016;7(2):27-31.
