# Supplementary material for: An endothelial SOX18–mevalonate pathway axis enables repurposing of statins for infantile hemangioma
Source: J Clin Invest. 2025 Feb 25;135(7):e179782. doi: 10.1172/JCI179782 (PMC11957709; doi:10.1172/JCI179782)
Supplement: Unedited blot and gel images [file jci-135-179782-s269.pdf]

Figure 3 B, D, F Western Blots for full tiffs rev 11/26/24

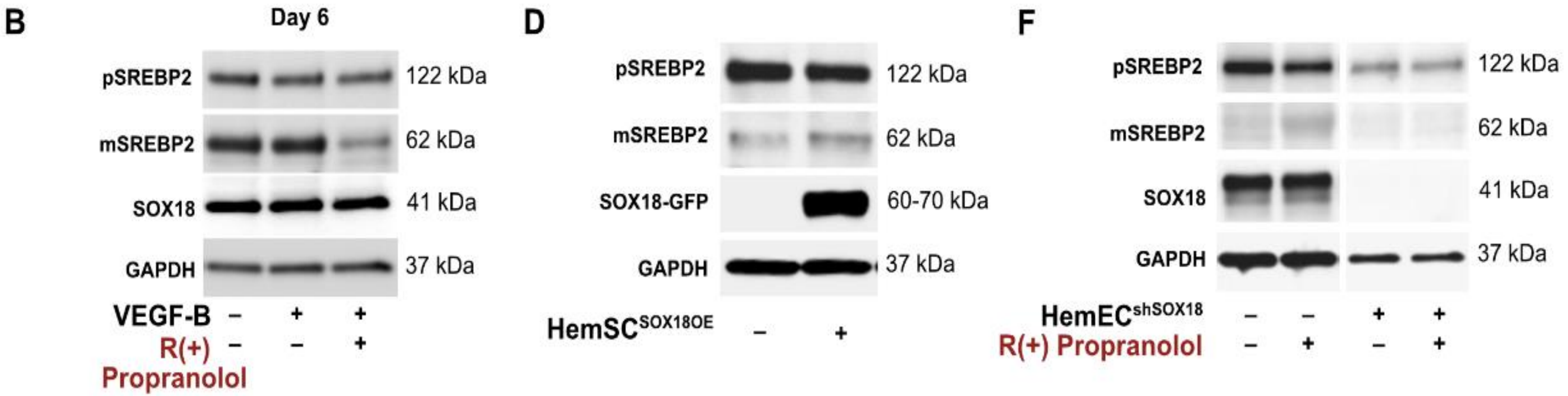

Figure 3 B Western Blots for full tiffs rev 11/26/24

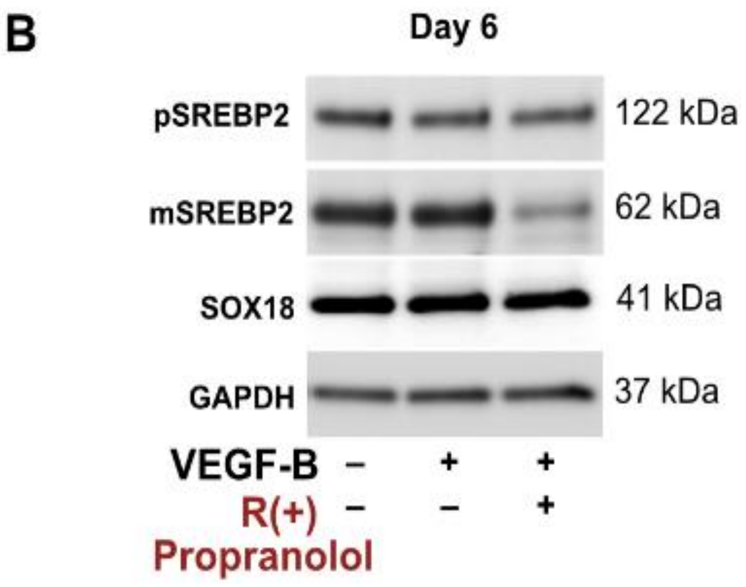

SREBP2

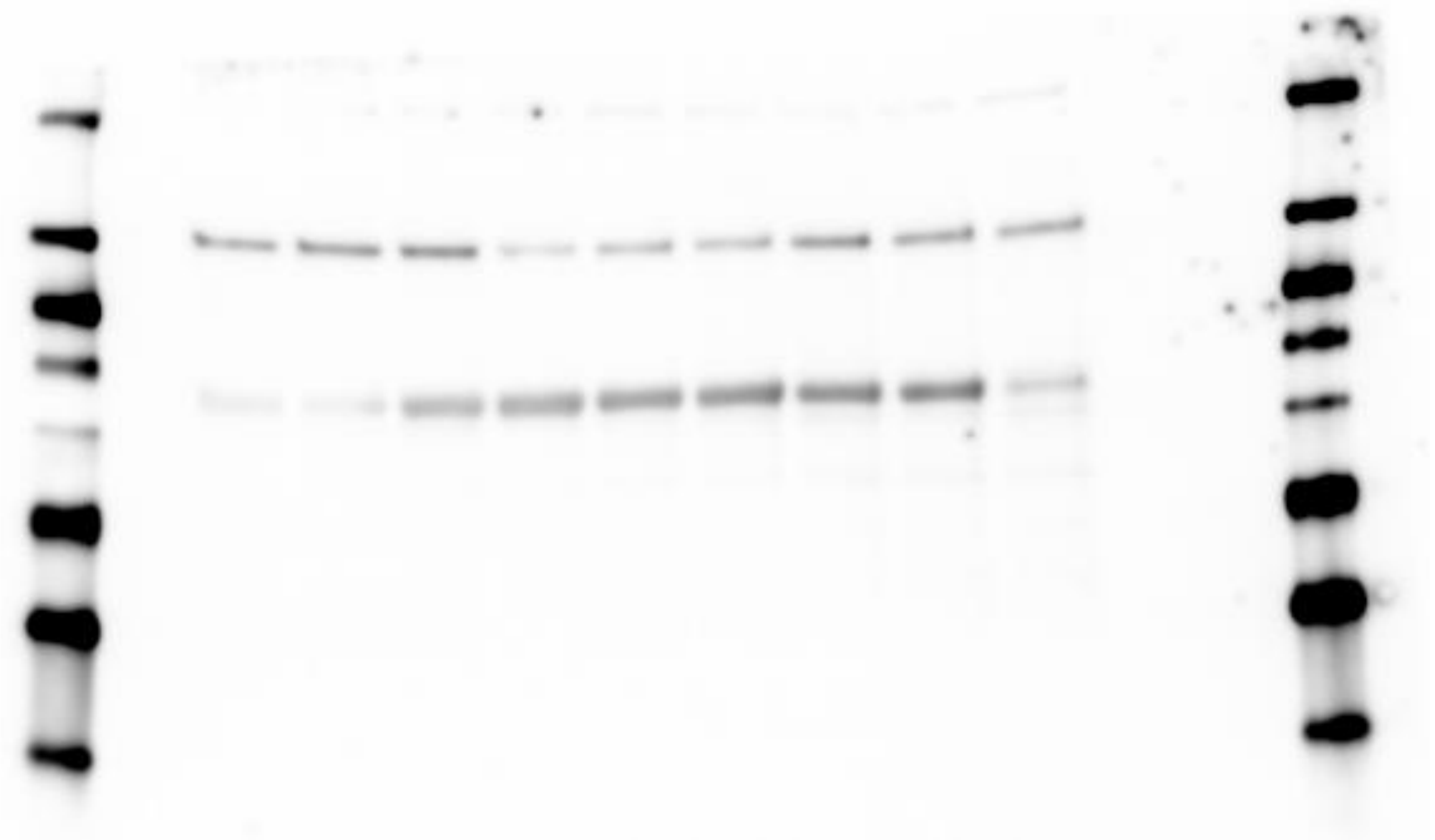

Tiff name: 042123 HemSC149 Differentiation 2hr R+ Treatments SREBP2 7m_PUB_600
Location: G:\Shared drives\SR-Bischoff-Common\Jill Wylie-Sears\For Anne\Figure 3 Find full tiffs 11262024\Figure 3 B Luke's HemSC149 SREBP2 2 hrs R+
and original location:
G:\Shared drives\SR-Bischoff-Common\Luke Borgelt\Western Blot\042123 HemSC149 Differentiation 2hr R+ treated samples SREBP2 WB\042123 HemSC149 Differentiation 2hr R+ Treatments SREBP2 WB

Figure 3 B Western Blots for full tiffs rev 11/26/24

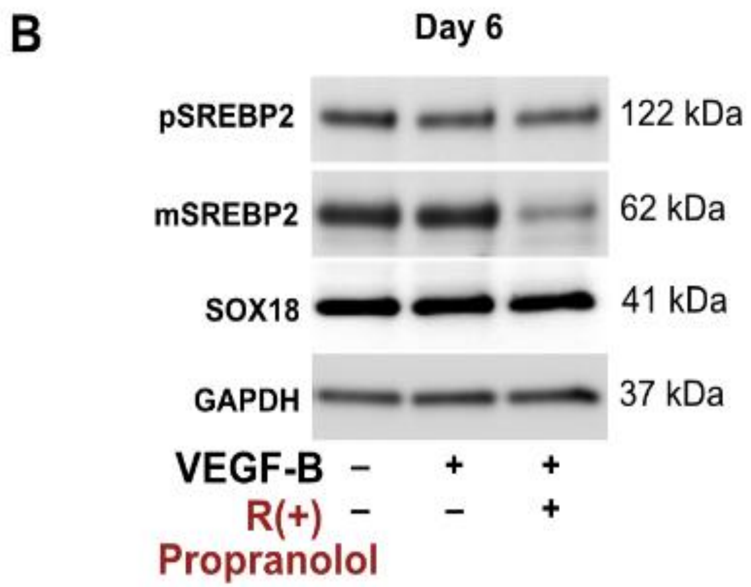

Sox18

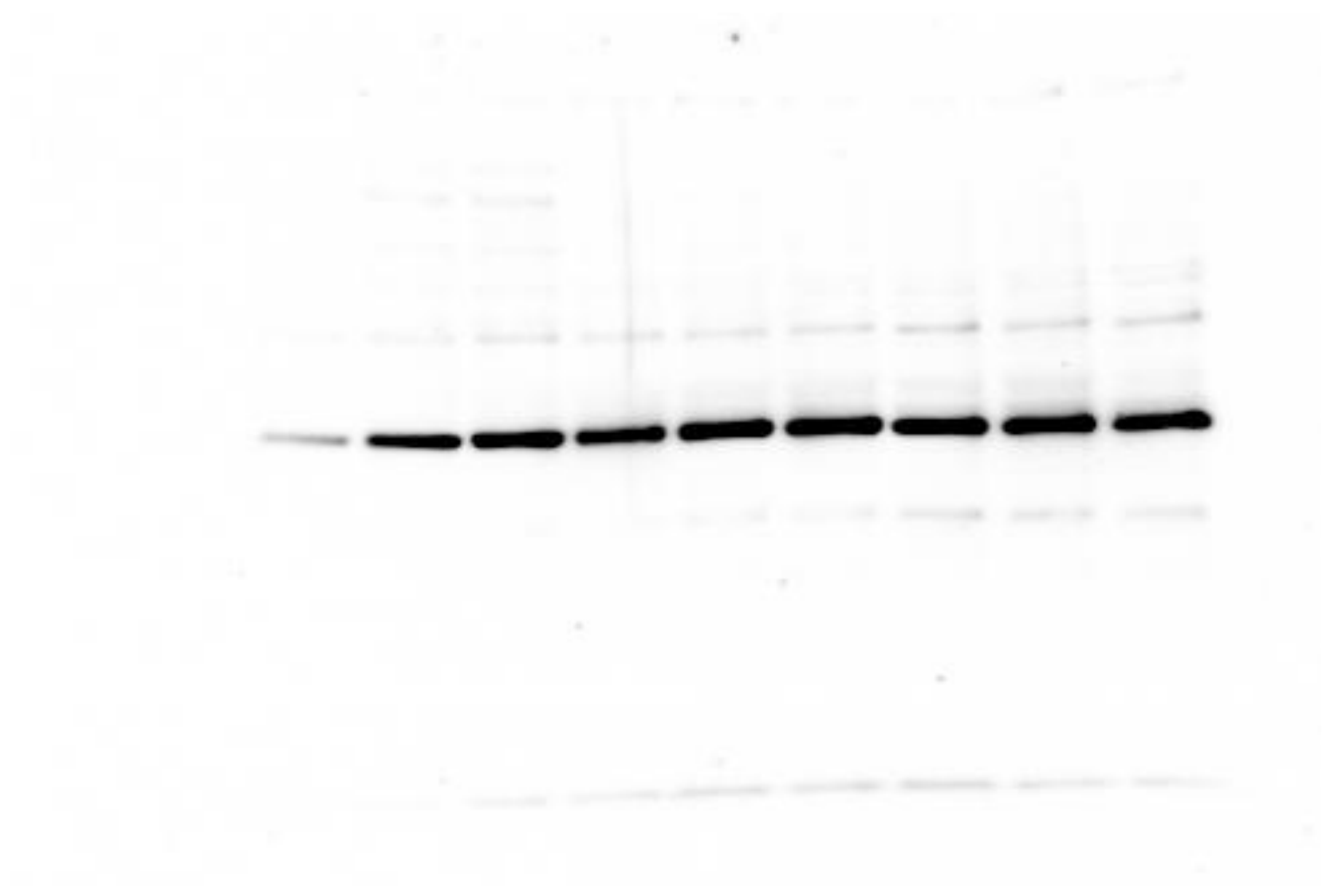

Tiff name: Sox18 cropped R+ 2 hrs 24.03.21_14.27.15_PUB_600
Location: G:\Shared drives\SR-Bischoff-Common\Jill Wylie-Sears\For Anne\Figure 3 Find full tiffs 11262024\Figure 3 B Luke's HemSC149 SREBP2 2 hrs R+

Figure 3 B Western Blots for full tiffs rev 11/26/24

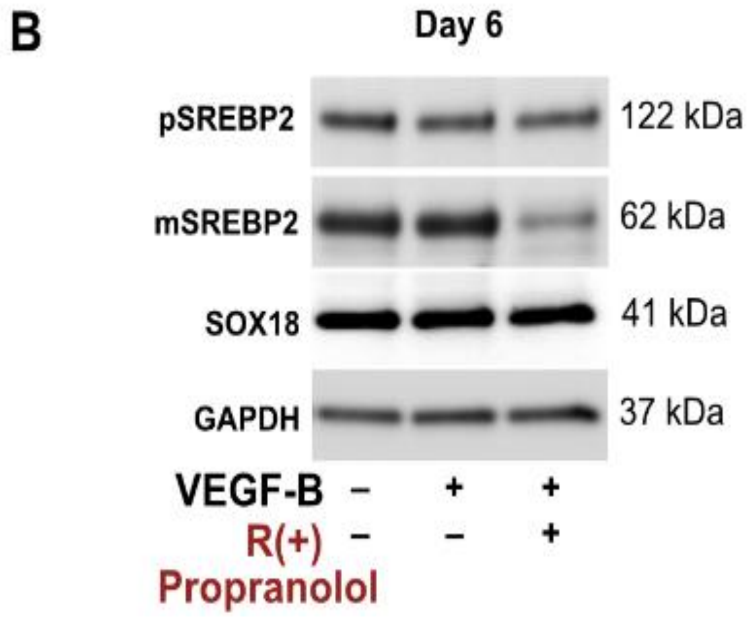

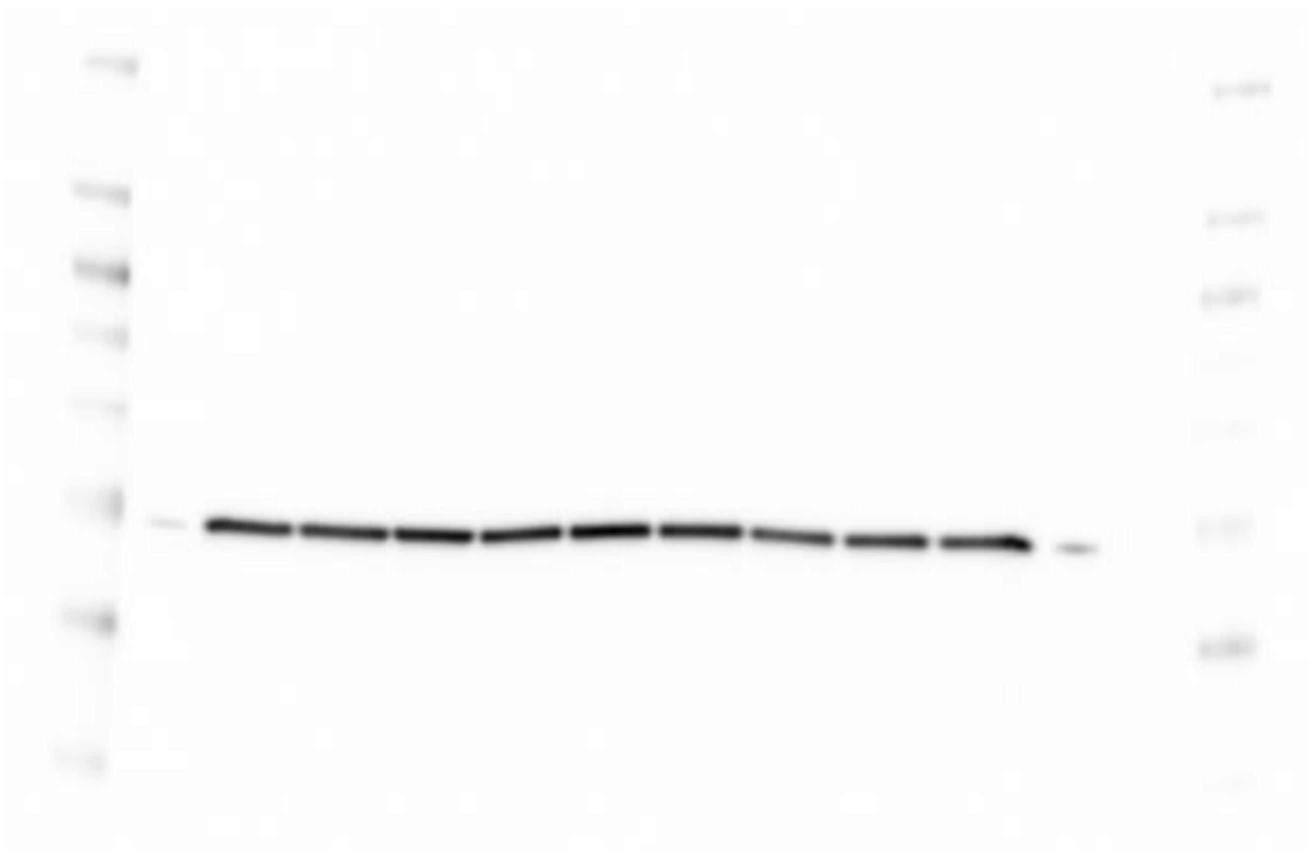

GAPDH

Tiff name: 042123 HemSC149 Differentiation 2hr R+ Treatments SREBP2 GAPDH 10s_PUB_600
Location: G:\Shared drives\SR-Bischoff-Common\Jill Wylie-Sears\For Anne\Figure 3 Find full tiffs 11262024\Figure 3 B Luke's HemSC149 SREBP2 2 hrs R+
and original location:
G:\Shared drives\SR-Bischoff-Common\Luke Borgelt\Western Blot\042123 HemSC149 Differentiation 2hr R+ treated samples SREBP2 WB\042123 HemSC149 Differentiation 2hr R+ Treatments SREBP2 WB

Figure 3 B Western Blots for full tiffs rev 11/26/24

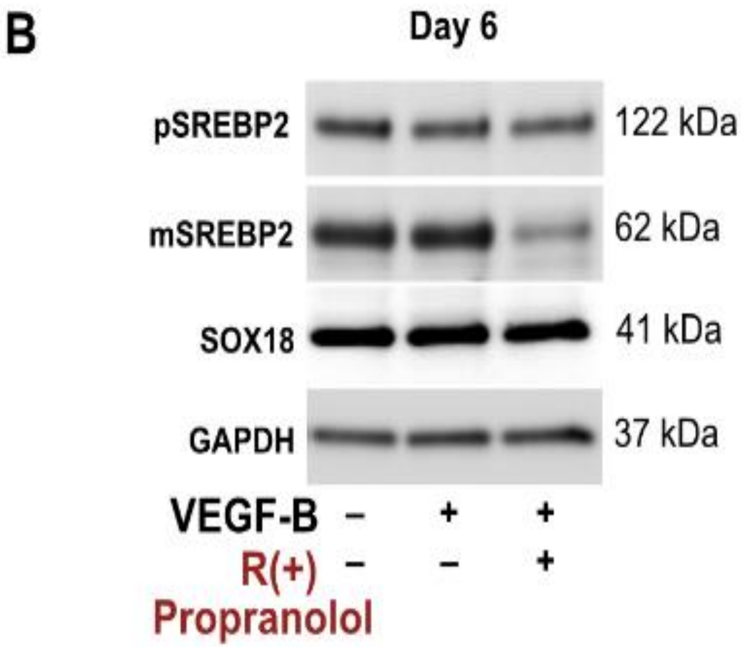

Tubulin

Tubulin
50 kDa

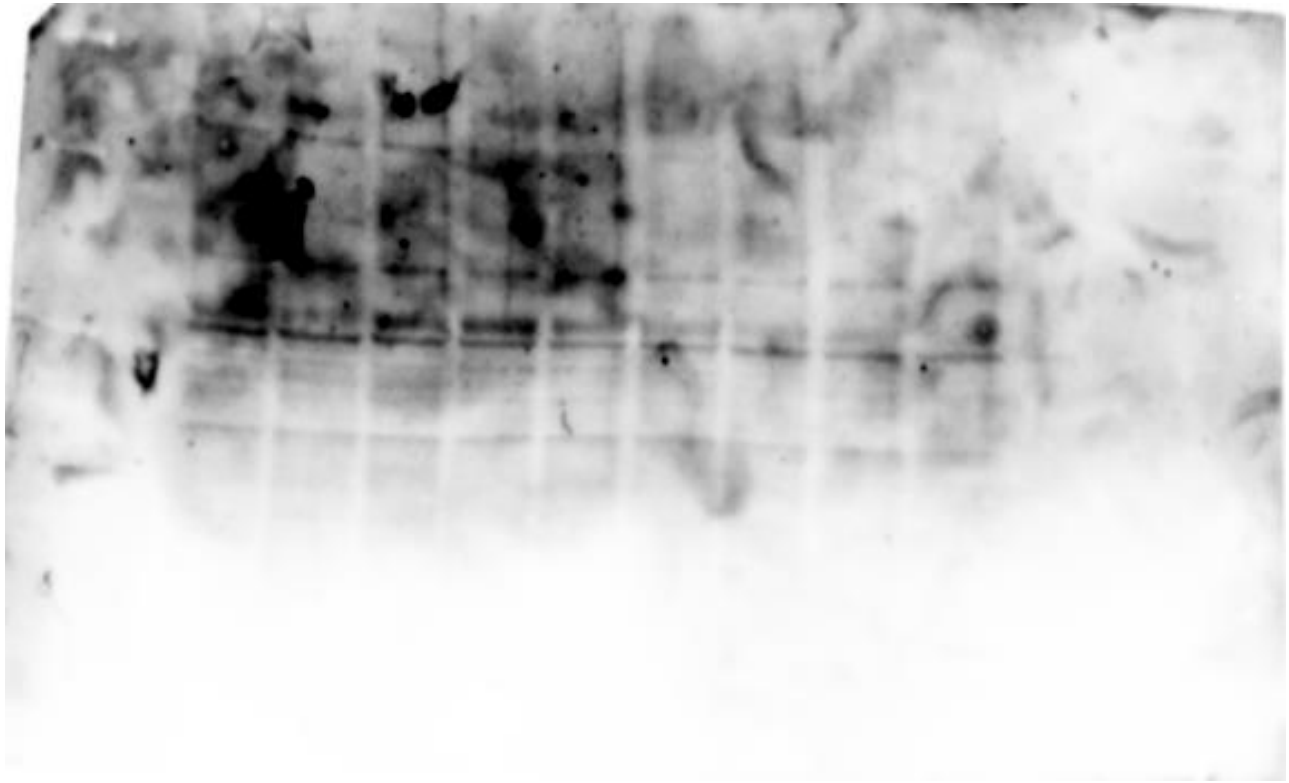

Tiff name: For Slide Tubulin B1 lighter crop 4m49s574ms 24.04.27_16.53.25_PUB_600
Location: G:\Shared drives\SR-Bischoff-Common\Jill Wylie-Sears\For Anne\Figure 3 Find full tiffs 11262024\Figure 3 B Luke's HemSC149 SREBP2 2 hrs R+

Figure 3 B Western Blots for full tiffs rev 11/26/24

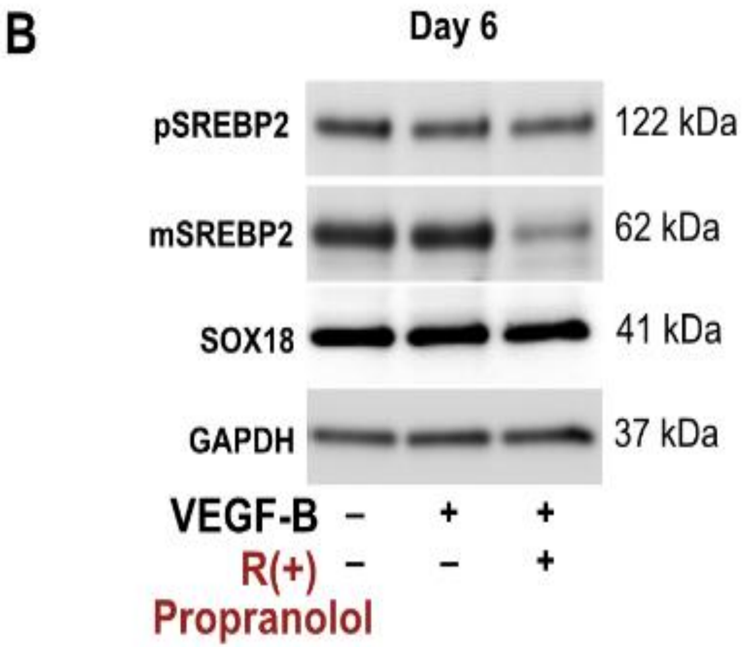

Tubulin
50 kDa

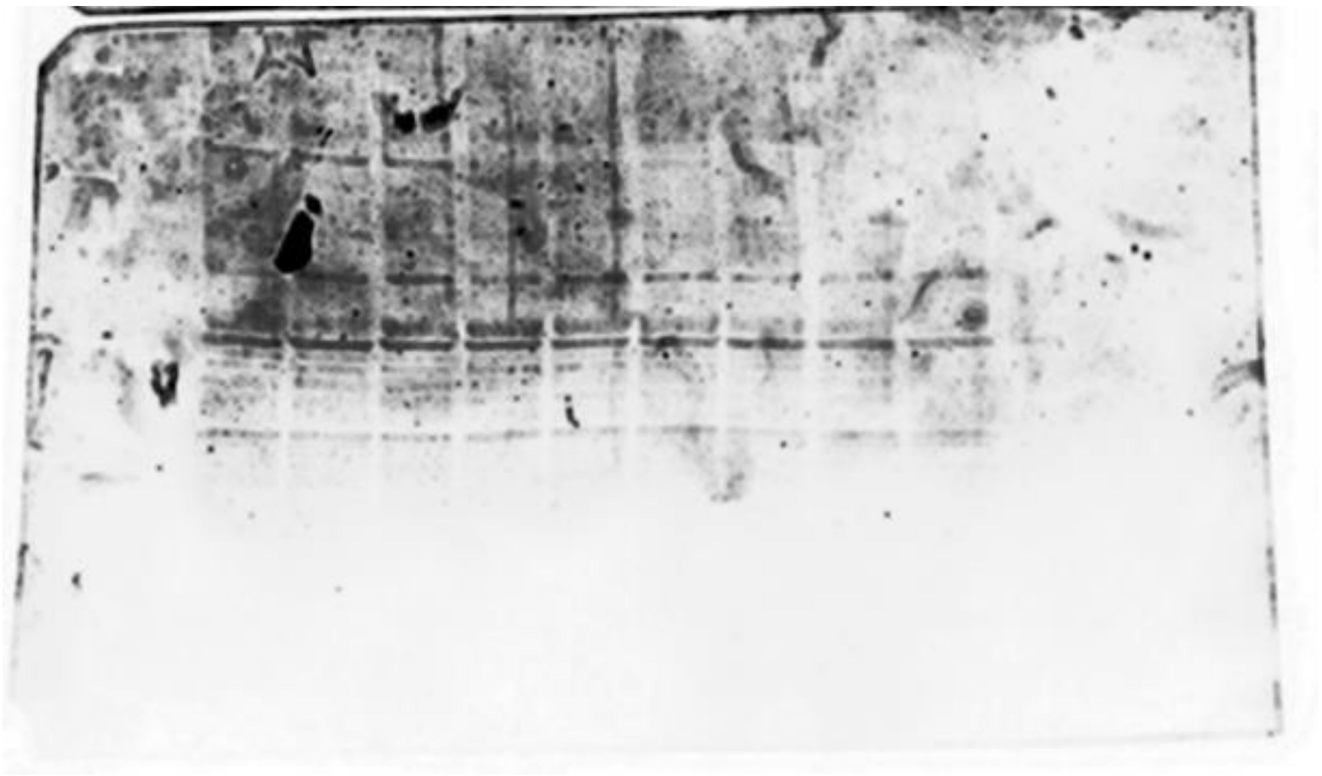

Tubulin

Tiff name: For Slide 2hrs R+ Tubulin 4m wide dynamic crop 24.04.27_15
Location: G:\Shared drives\SR-Bischoff-Common\Jill Wylie-Sears\For Anne\Figure 3 Find full tiffs 11262024\Figure 3 B Luke's HemSC149 SREBP2 2 hrs R+

HemSC149 p8 Differentiation R+ 2hr treatments

Luke Borgelt

04/21/23

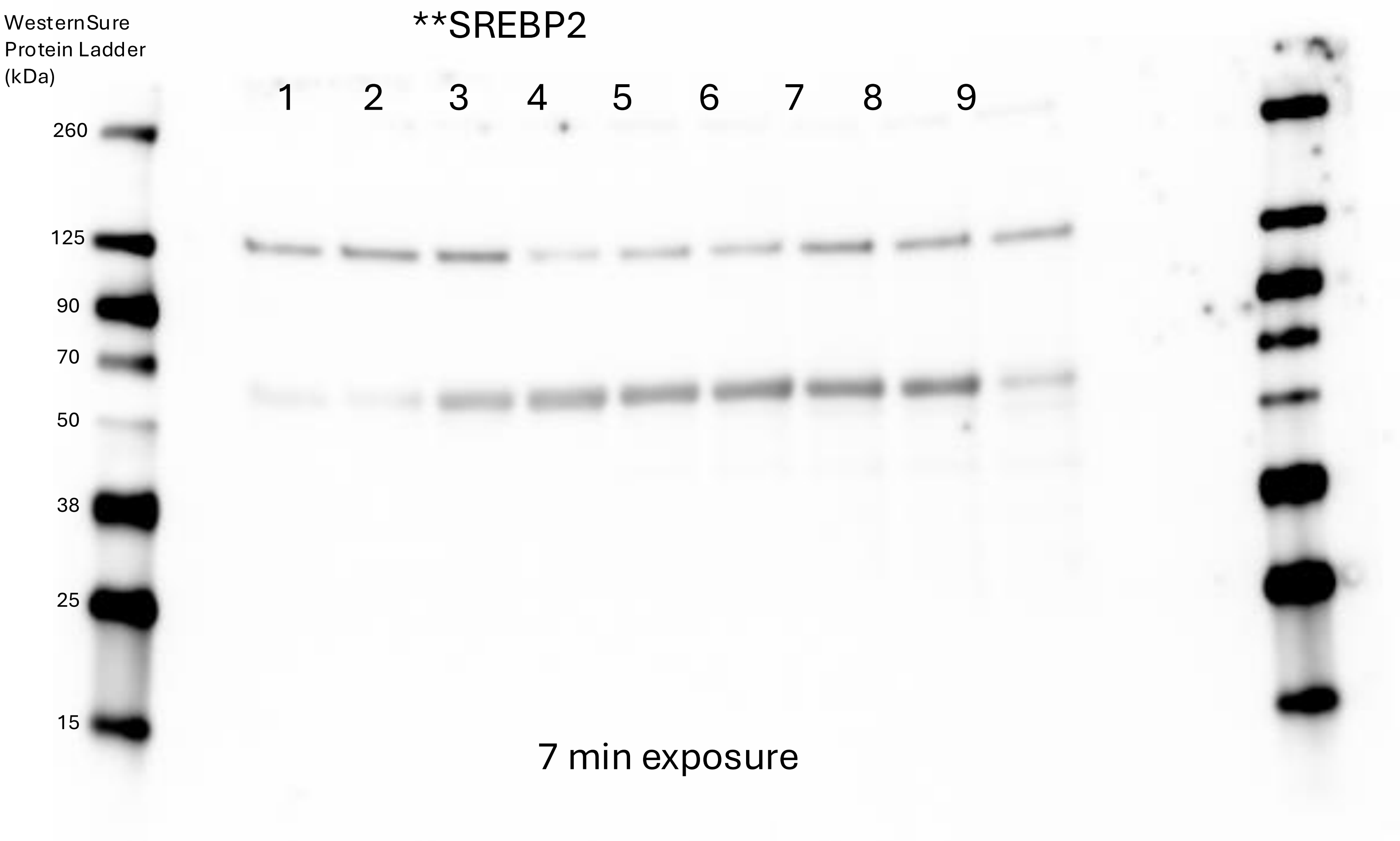

Lanes:

1- D2 –VEGFB, + 0.01% HCl (VEGFB vehicle)
2- D2 +VEGFB, 0.01% ddH2O (R+ vehicle)
3- D2 +VEGFB, 20uM R+ (2hrs)
4- D4 –VEGFB, + 0.01% HCl (VEGFB vehicle)
5- D4 +VEGFB, 0.01% ddH2O (R+ vehicle)
6- D4 +VEGFB, 20uM R+ (2hrs)
7- D6 –VEGFB, + 0.01% HCl (VEGFB vehicle)
8- D6 +VEGFB, 0.01% ddH2O (R+ vehicle)
9- D6 +VEGFB, 20uM R+ (2hrs)

Sox18 Western Blot - HemSC149 p8 Differentiation R+ 2hr treatments (LB 4/21/23) reprobed 3/21/24 JWS

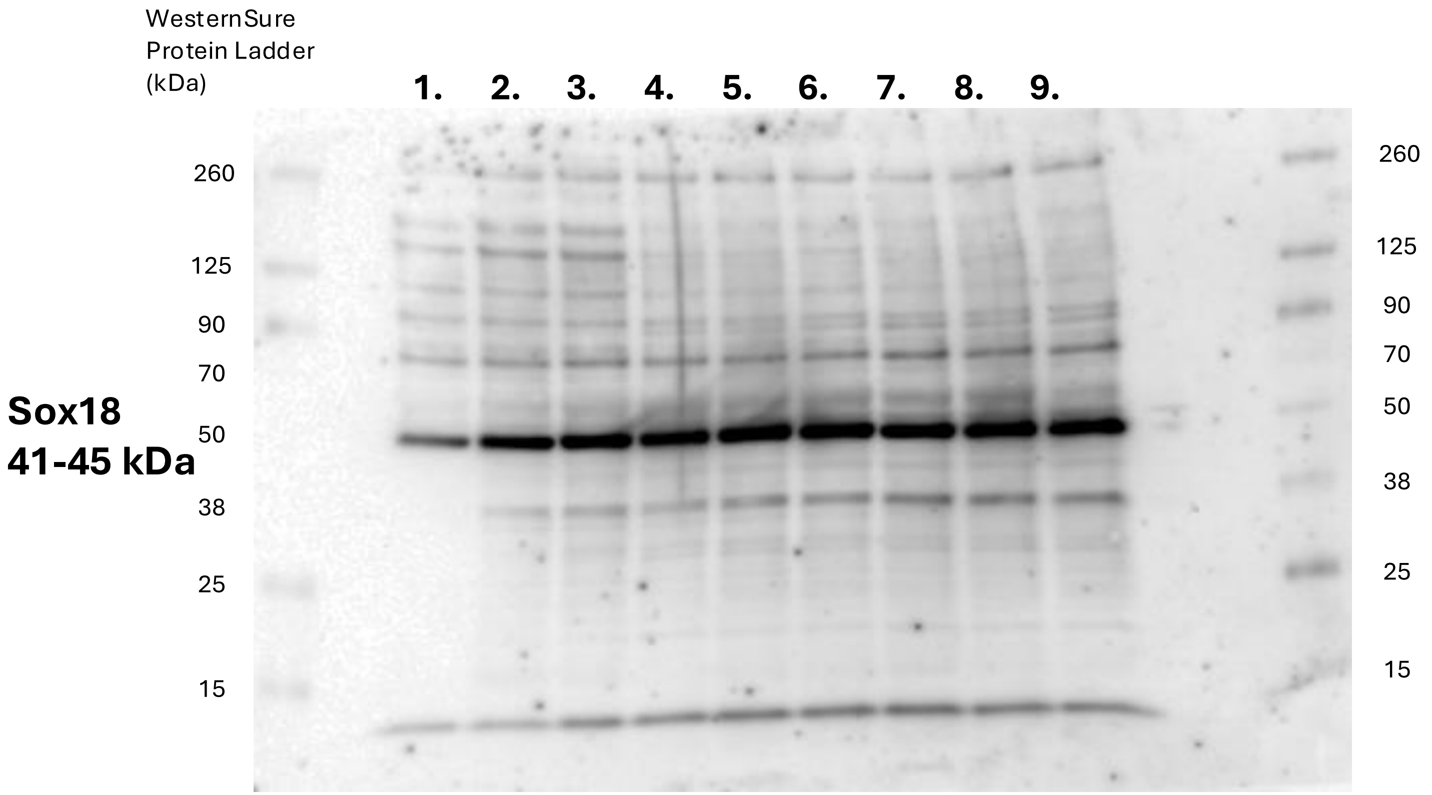

1 min Azure Imager

1. D2 Control
2. D2 VEGFB
3. D2 R+ (2hrs)
4. D4 Control
5. D4 VEGB
6. D4 R+ 2hrs
7. D6 Control
8. D6 VEGFB
9. D6 R+ 2hrs

30 ug protein lysate/well

Block 1 hour RT in 5% Milk/TBS-T

Mouse anti-human Sox18 (1:500)
Incubation overnight at 4C

2nd mouse HRP (1:2000)
Incubation 1 hour at room temperature (RT)

All antibodies in 5% Milk/TBS-T

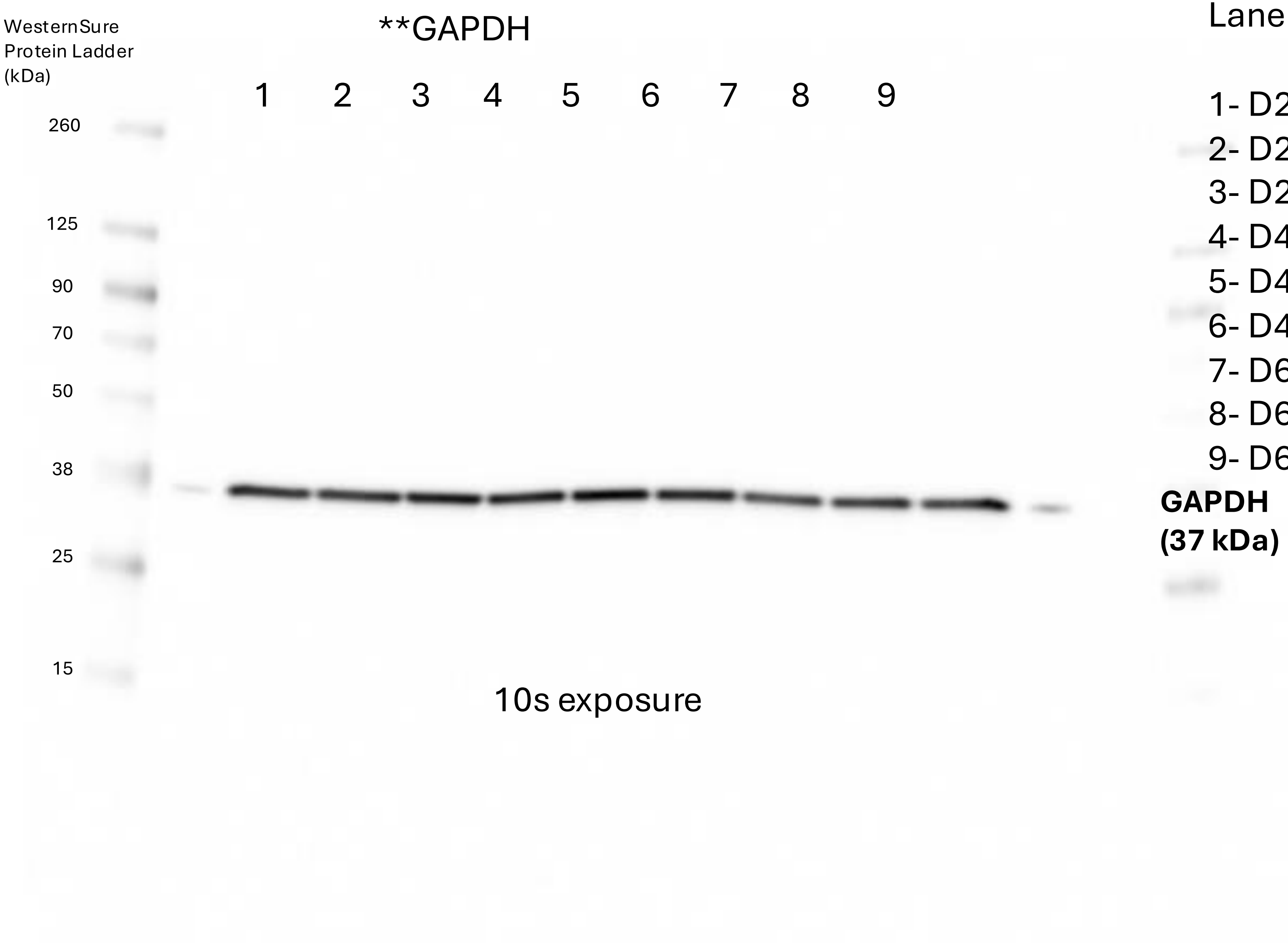

Lanes:

1- D2 –VEGFB, + 0.01% HCl (VEGFB vehicle)
2- D2 +VEGFB, 0.01% ddH2O (R+ vehicle)
3- D2 +VEGFB, 20uM R+ (2hrs)
4- D4 –VEGFB, + 0.01% HCl (VEGFB vehicle)
5- D4 +VEGFB, 0.01% ddH2O (R+ vehicle)
6- D4 +VEGFB, 20uM R+ (2hrs)
7- D6 –VEGFB, + 0.01% HCl (VEGFB vehicle)
8- D6 +VEGFB, 0.01% ddH2O (R+ vehicle)
9- D6 +VEGFB, 20uM R+ (2hrs)

30 ug protein lysate/well

GAPDH **37 kDa**

Tubulin* Western Blot - HemSC149 p8 Differentiation R+ 2hr treatments (LB 4/21/23) reprobed 4/26/24 JWS

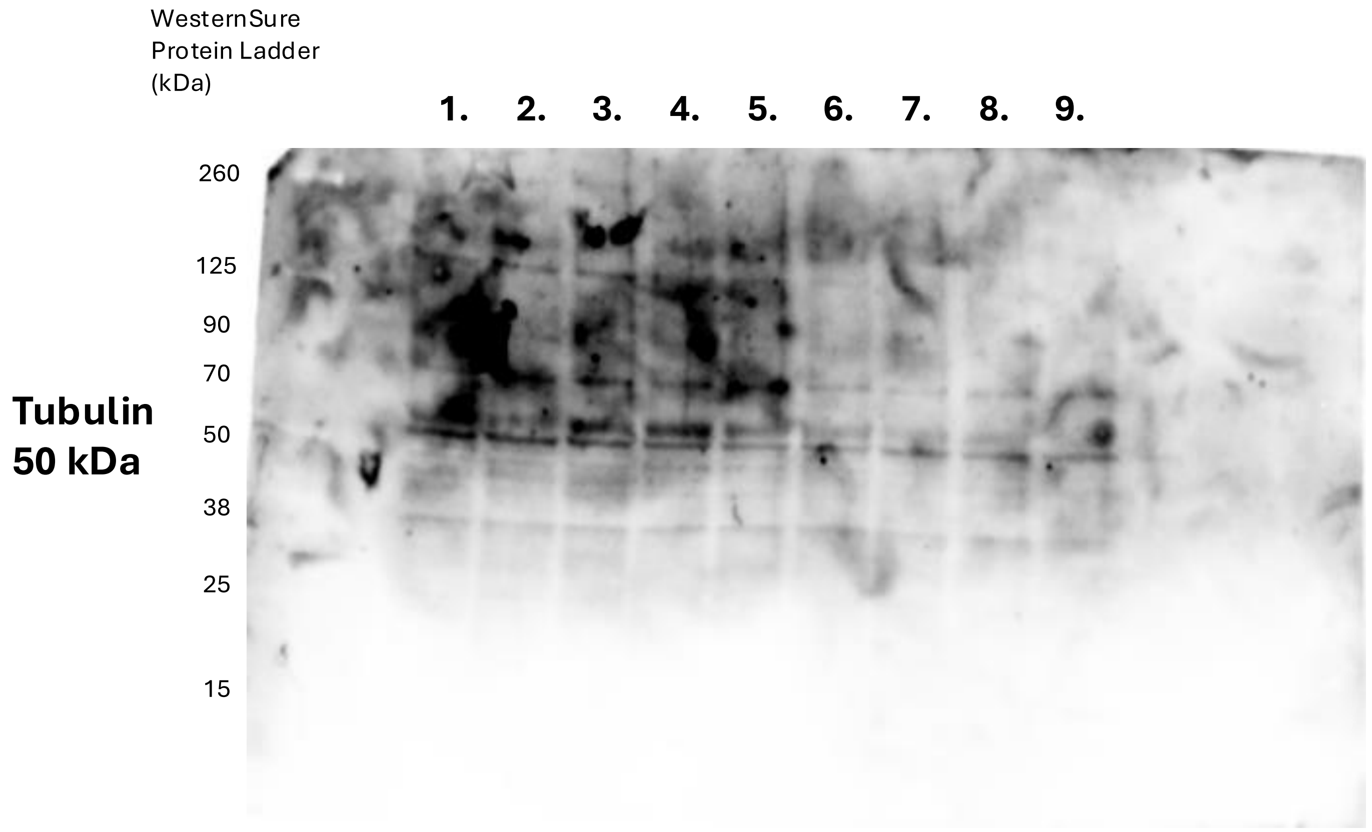

1 min Azure Imager

1. D2 Control
2. D2 VEGFB
3. D2 R+ (2hrs)
4. D4 Control
5. D4 VEGB
6. D4 R+ 2hrs
7. D6 Control
8. D6 VEGFB
9. D6 R+ 2hrs

30 ug protein lysate/well
Block 1 hour RT in 5% Milk/TBS-T
Goat anti-mouse Tubulin (1:2000)
Incubation overnight at 4C
2nd goat HRP (1:2000)
Incubation 1 hour at room temperature (RT)
All antibodies in 5% Milk/TBS-T

*Recombinant Anti-alpha Tubulin (acetyl K40) antibody [EPR16772] - Goat IgG (Chimeric) (ab289875) reacts with mouse, rat, human
This goat monoclonal chimeric antibody has been engineered from a RabMAb parent antibody (ab179484).
By necessity, some rabbit sequence is retained as part of the variable domain.
https://www.abcam.com/products/primary-antibodies/alpha-tubulin-acetyl-k40-antibody-epr16772-goat-igg-chimeric-ab289875.html

Tubulin* Western Blot - HemSC149 p8 Differentiation R+ 2hr treatments (LB 4/21/23) reprobed 4/26/24 JWS

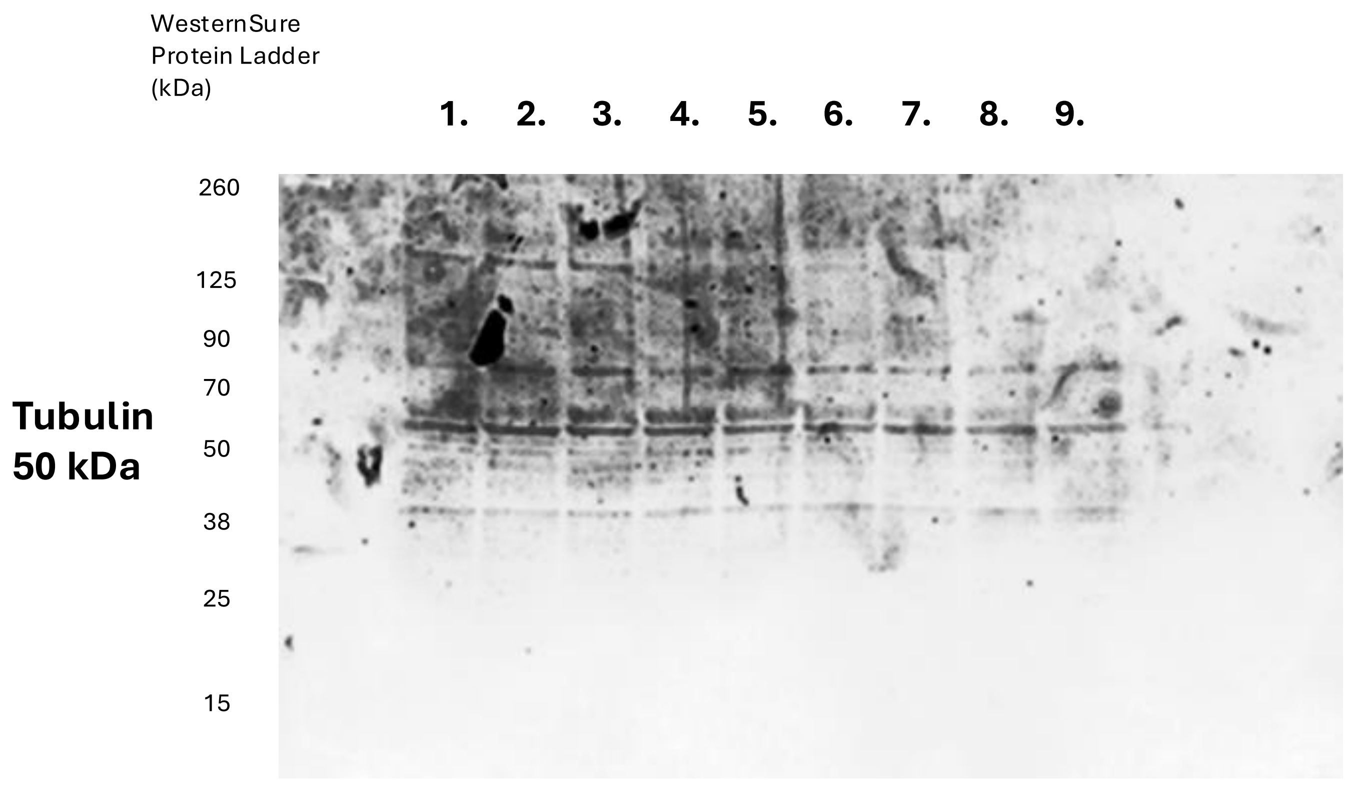

4 min less overall gamma Wide Dynamic Azure

1. D2 Control
2. D2 VEGFB
3. D2 R+ (2hrs)
4. D4 Control
5. D4 VEGB
6. D4 R+ 2hrs
7. D6 Control
8. D6 VEGFB
9. D6 R+ 2hrs

30 ug protein lysate/well
Block 1 hour RT in 5% Milk/TBS-T
Goat anti-mouse Tubulin (1:2000)
Incubation overnight at 4C
2nd goat HRP (1:2000)
Incubation 1 hour at room temperature (RT)
All antibodies in 5% Milk/TBS-T

*Recombinant Anti-alpha Tubulin (acetyl K40) antibody [EPR16772] - Goat IgG (Chimeric) (ab289875) reacts with mouse, rat, human
This goat monoclonal chimeric antibody has been engineered from a RabMAb parent antibody (ab179484).
By necessity, some rabbit sequence is retained as part of the variable domain.
https://www.abcam.com/products/primary-antibodies/alpha-tubulin-acetyl-k40-antibody-epr16772-goat-igg-chimeric-ab289875.html

HemSC149 p8 EC Differentiation Assay, R+ Propranolol (20 uM) 2 hours, 04/21/23 LB, reprobe Sox18 03/21/24 JWS

Lanes:

1. D2 (–)VEGFB, + 0.01% HCl (VEGFB vehic
2. D2 +VEGFB, 0.01% ddH2O (R+ vehicle)
3. D2 +VEGFB, 20uM R+ (2hrs)
4. D4 (–)VEGFB, + 0.01% HCl (VEGFB vehic
5. D4 +VEGFB, 0.01% ddH2O (R+ vehicle)
6. D4 +VEGFB, 20uM R+ (2hrs)
7. D6 (–)VEGFB, + 0.01% HCl (VEGFB vehic
8. D6 +VEGFB, 0.01% ddH2O (R+ vehicle)
9. D6 +VEGFB, 20uM R+ (2hrs)

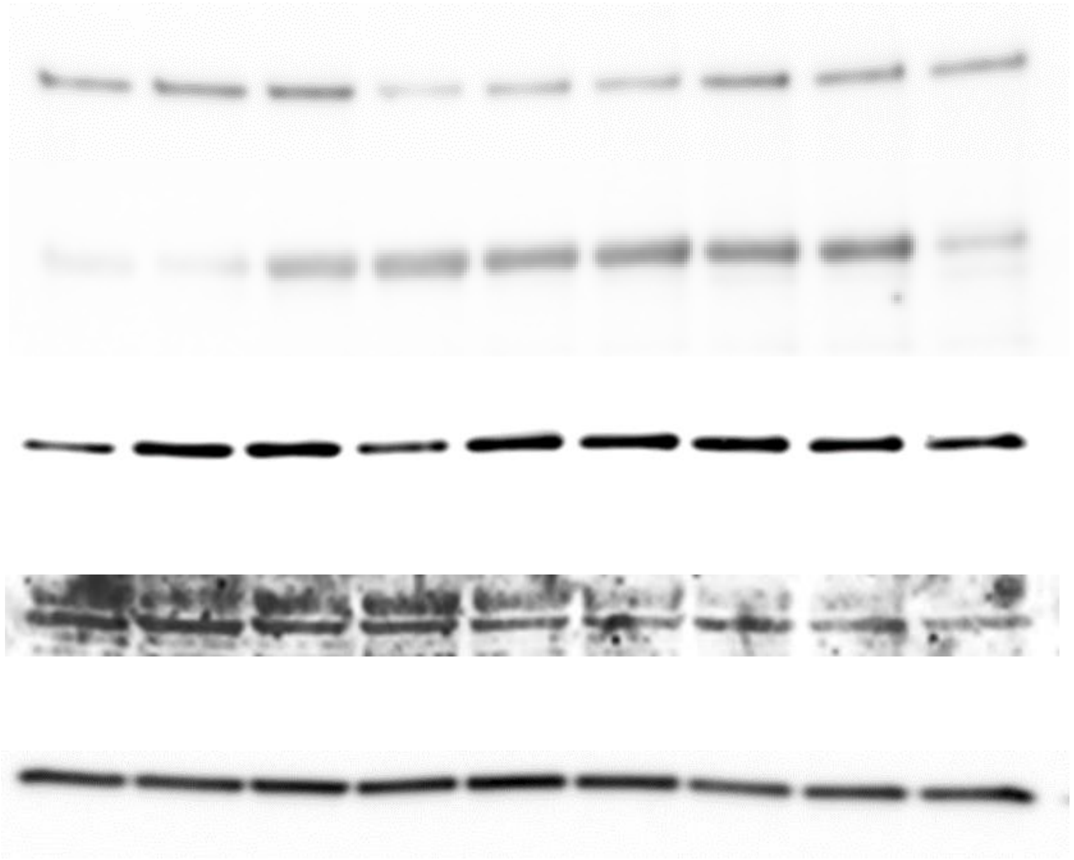

SREBP2 (122 kDa band)

SREBP2 (55-65 kDa band)

Sox18 (41-45 kDa band)

(Remnant SREBP2 (60 kDa) above)
Tubulin (50 kDa)

GAPDH (37 kDa band)

1 2 3 4 5 6 7 8 9

Figure 3 D Western Blots for full tiffs rev 11/26/24

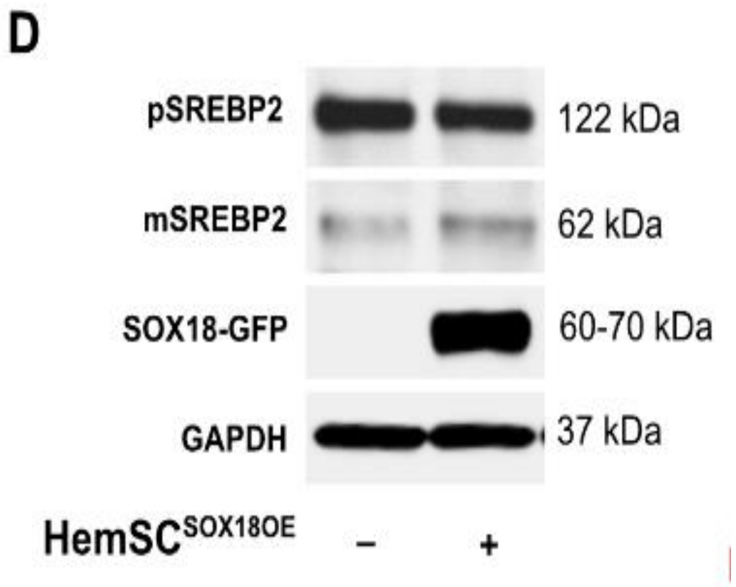

SREBP2

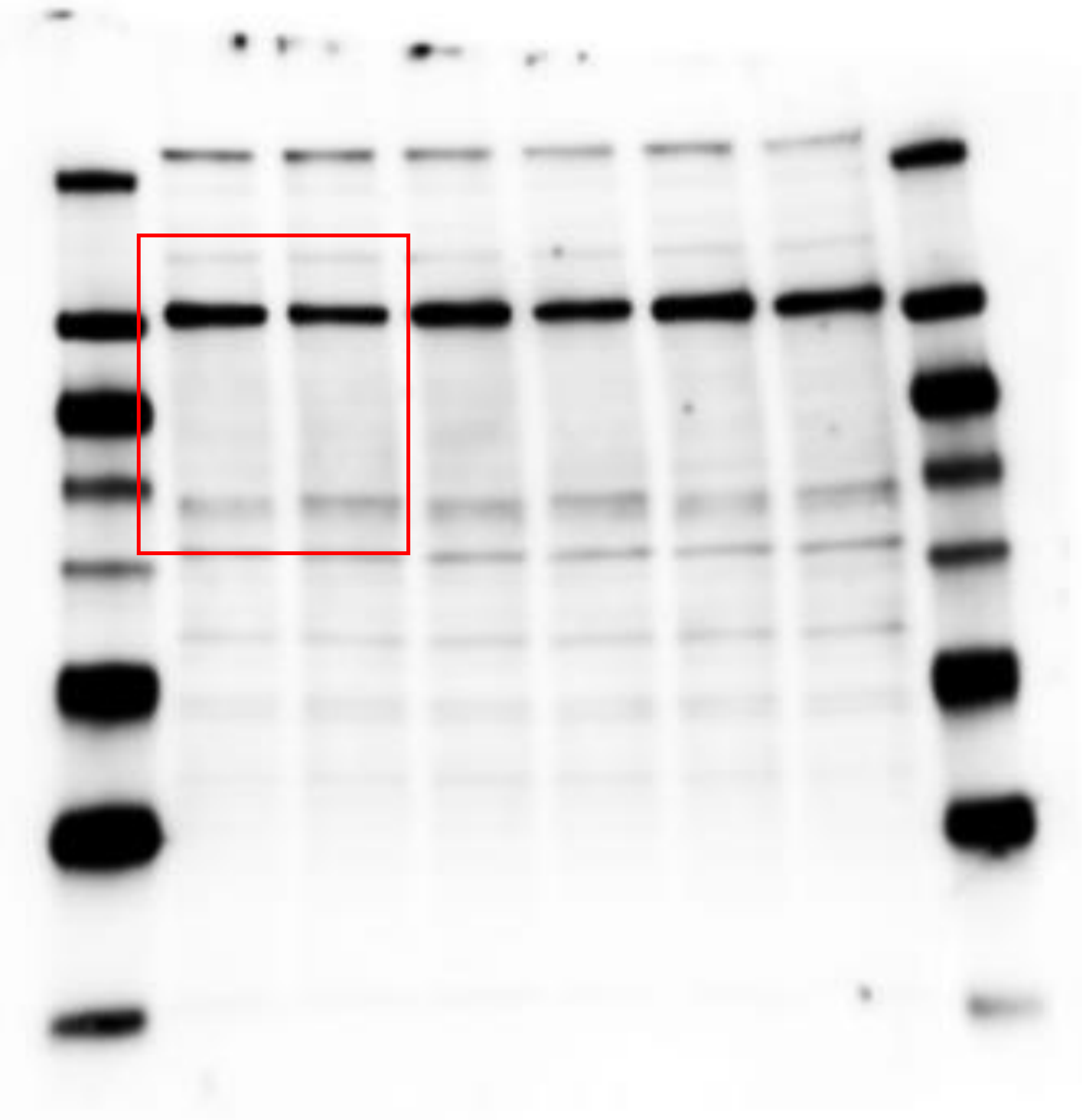

Tiff name: 8 bit SREBP2 3 min sharp auto straight crop 24.09.01_15.14.57_PUB_600
Location: G:\Shared drives\SR-Bischoff-Common\Jill Wylie-Sears\For Anne\Figure 3 Find full tiffs 11262024\Figure 3 D Sox18 OE HemSC SREBP2 Sox18GFP GAPDH

Figure 3 D Western Blots for full tiffs rev 11/26/24

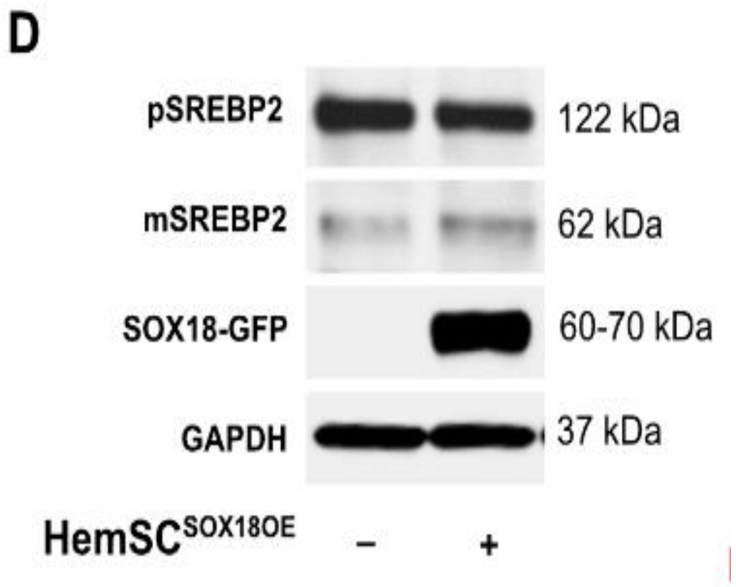

Sox18, GAPDH

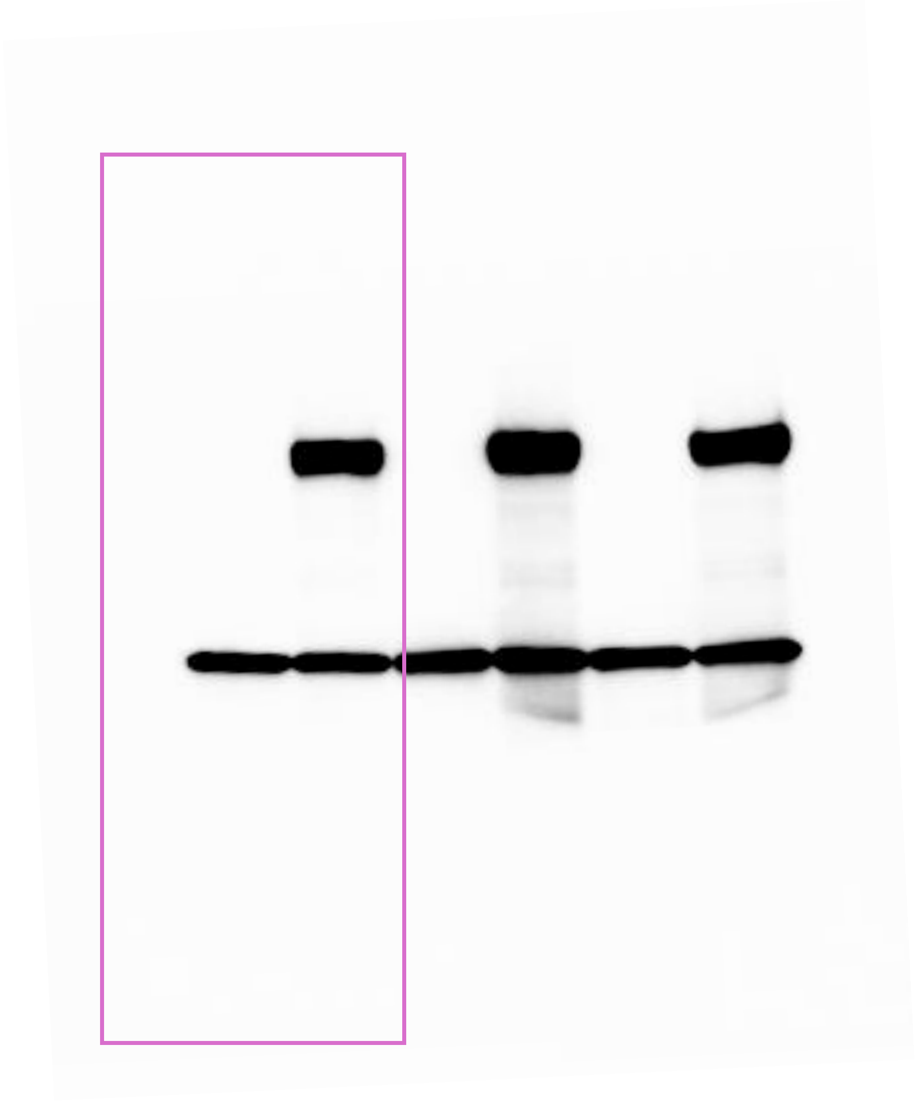

Tiff name: 420ms autoexposure more gamma 24.08.10_15.16.11_PUB_600
Location: G:\Shared drives\SR-Bischoff-Common\Jill Wylie-Sears\For Anne\Figure 3 Find full tiffs 11262024\Figure 3 D Sox18 OE HemSC SREBP2 Sox18GFP GAPDH

SREBP2 precursor, mature forms – 2 Days, HemSC171 ev, Sox18 Overexpression (triplicates) in EGM-2 Complete media 8/9/24
5uM ALLN in RIPA proteinase inhibitors cocktail AND 2 hours 100 uM ALLN pre-treatment (following Luke's 4/21/23 protocol) - triplicates

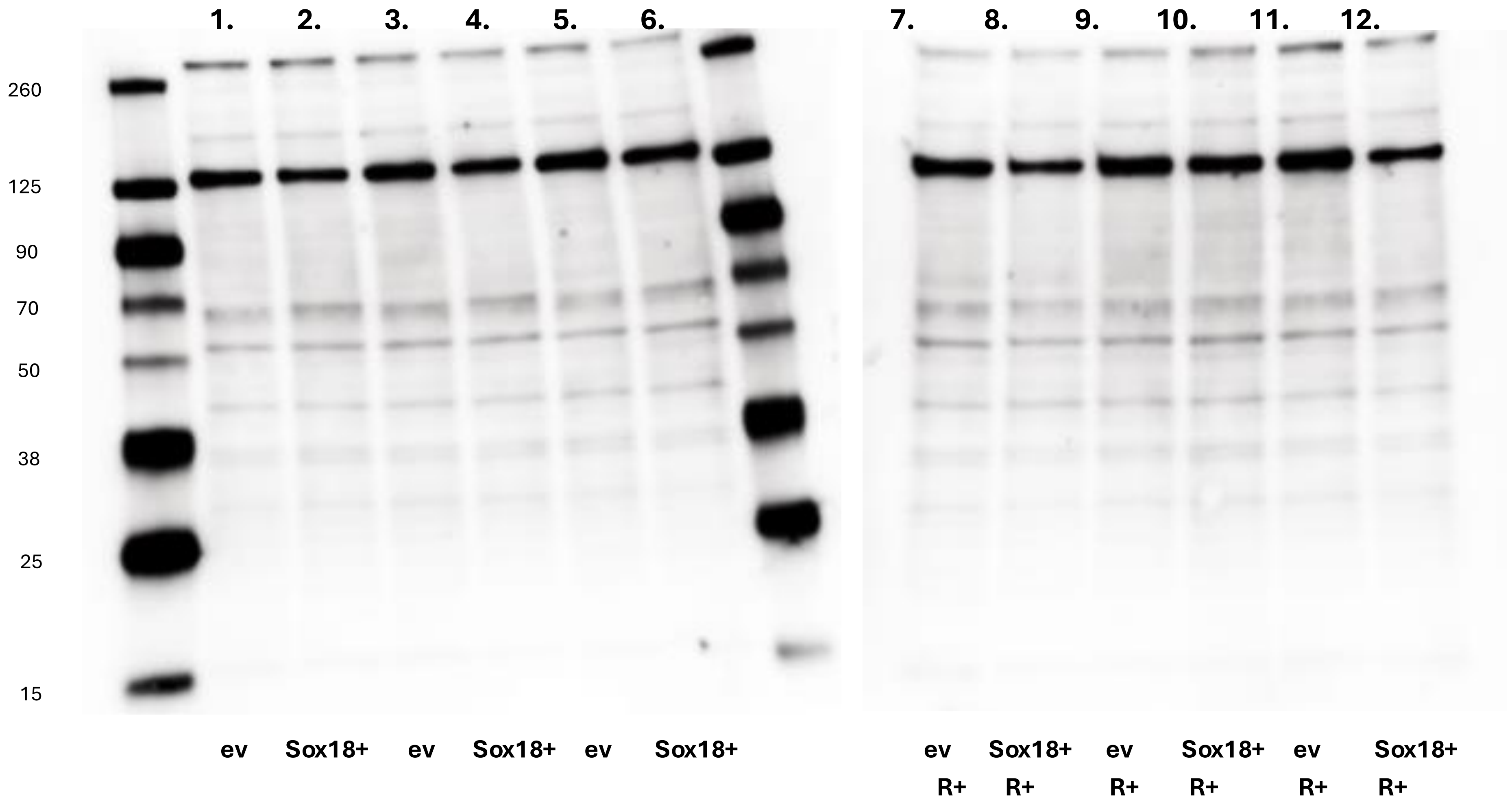

Wide Dynamic (1m10s822ms) Azur
more overall gamma
SREBP2 precursor, SREBP2 mature
1. HemSC ev (1)
2. HemSC Sox18 OE (1)
3. HemSC ev (2)
4. HemSC Sox18 OE (2)
5. HemSC ev (3)
6. HemSC Sox18 OE (3)
7. HemSC ev R+ Prop (1)
8. HemSC Sox18 OE R+ Prop (1)
9. HemSC ev R+ Prop (2)
10. HemSC Sox18 OE R+ Prop (2)
11. HemSC empty vector R+ Prop (3)
12. HemSC Sox18 OE R+ Prop (3)

30 ug protein lysate/well

Block 1 hour RT in 5% Milk/TBS-T

Rabbit anti-mouse SREBP2 (1:100)
Incubation overnight at 4C

2nd rabbit HRP (1:2000)
Incubation overnight at 4C
All antibodies in 5% Milk/TBS-T

***** Protein ladder lanes, very narrow and blurred into multiple lanes, did not show top 260 kDa band at all, which led to incorrect blot cut at top.***

<u>Old SREBP2 antibody in 5% Milk/TBS-T/0.02% Na Azide with SREBP2 Lot# 4120677 (MilliporeSigma Cat# MABS1988)</u>
Previous lots not available. **SREBP2 Precursor 122 kDa**
Mature form 55, 62 kDa

Sox18, GAPDH – 2 Days, HemSC171 ev, Sox18 Overexpression (triplicates) in EGM-2 Complete media 8/10/24
5uM ALLN in RIPA proteinase inhibitors cocktail AND 2 hours 100 uM ALLN pre-treatment (following Luke's 4/21/23 protocol) - triplicates

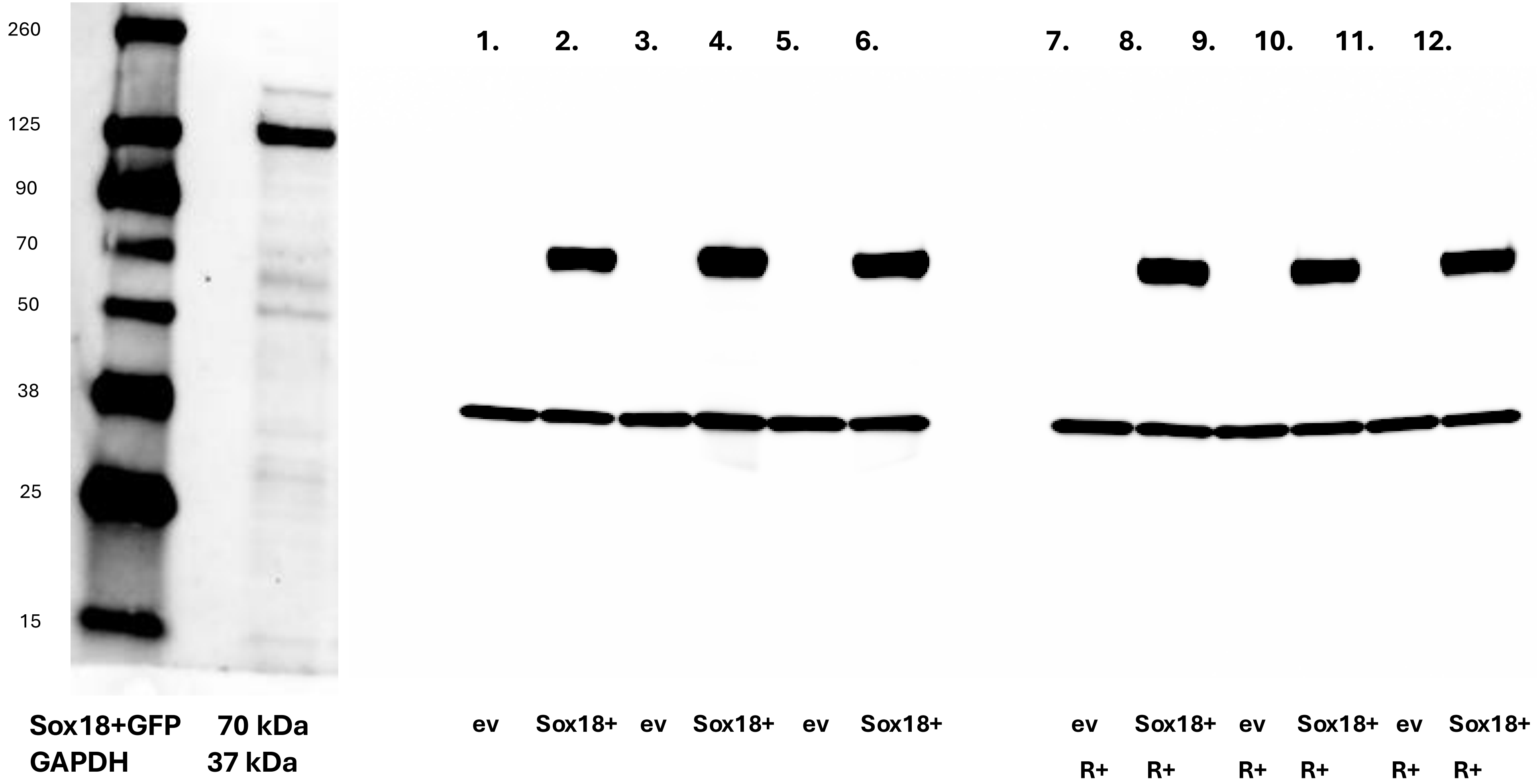

Sox18+GFP 70 kDa
GAPDH 37 kDa

*** This blot was previously incubated with SREBP2**

2 sec Azure Imager
less overall gamma

Sox18, GAPDH
1. HemSC ev (1)
2. HemSC Sox18 OE (1)
3. HemSC ev (2)
4. HemSC Sox18 OE (2)
5. HemSC ev (3)
6. HemSC Sox18 OE (3)
7. HemSC ev R+ Prop (1)
8. HemSC Sox18 OE R+ Prop (1)
9. HemSC ev R+ Prop (2)
10. HemSC Sox18 OE R+ Prop (2)
11. HemSC empty vector R+ Prop (3)
12. HemSC Sox18 OE R+ Prop (3)

30 ug protein lysate/well

Block 1 hour RT in 5% Milk/TBS-T

Mouse anti-human Sox18 (1:1000)
Rabbit anti-human GAPDH (1:3000)
Incubation overnight at 4C

2nd mouse HRP (1:3000)
2nd rabbit HRP (1:3000)
Incubation for 1 hour room temperature
All antibodies in 5% Milk/TBS-T

Figure 3 F Western Blots for full tiffs rev 11/26/24

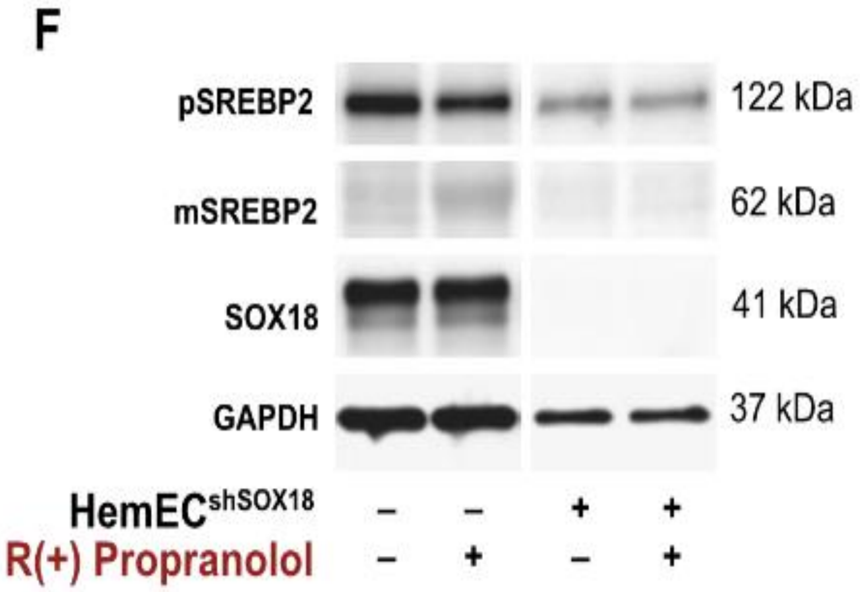

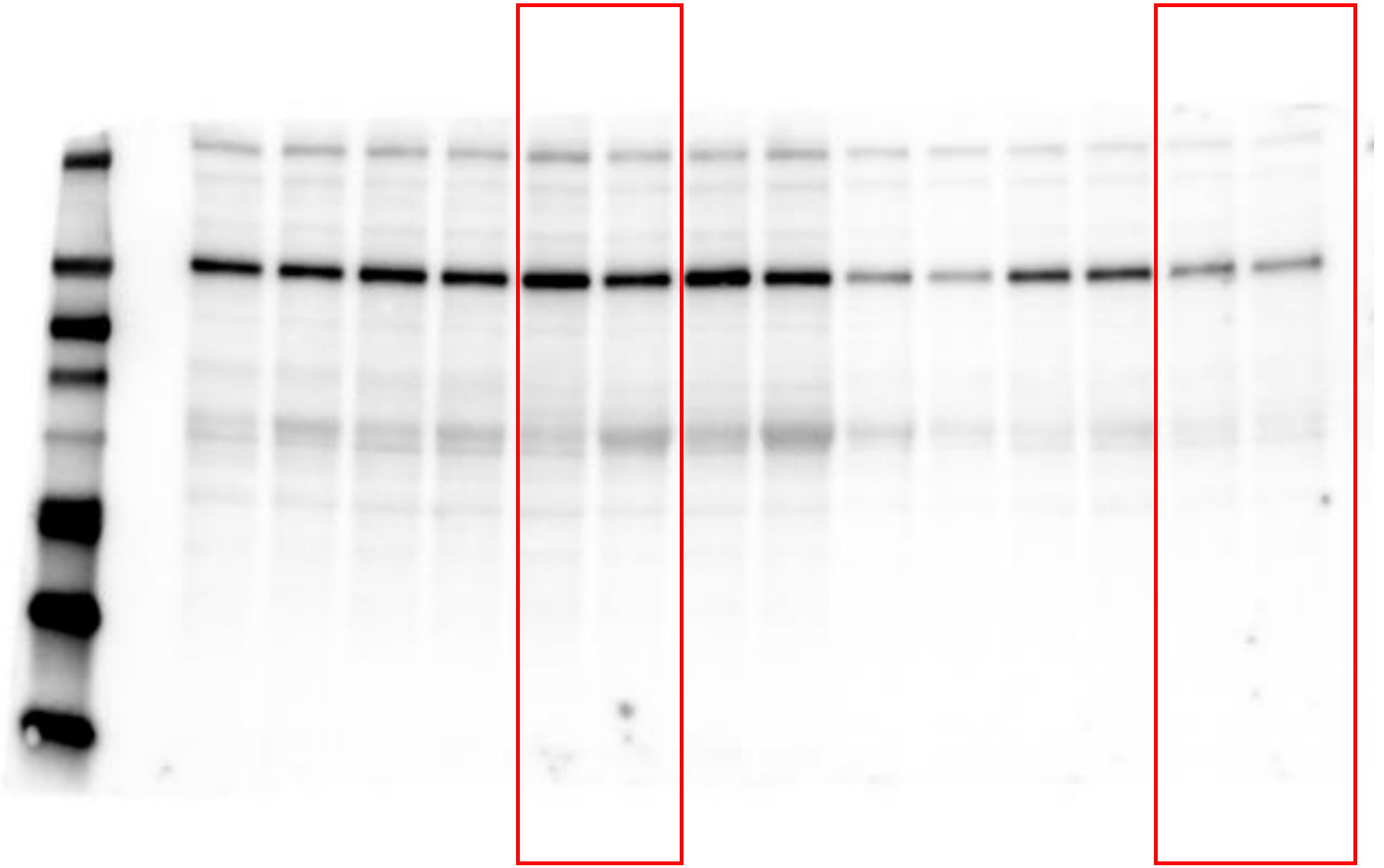

SREBP2

Tiff name: SREPB2 straightened 23.12.13_12.56.57_PUB_600
Location: G:\Shared drives\SR-Bischoff-Common\Jill Wylie-Sears\For Anne\Figure 3 Find full tiffs 11262024\Figure 3 F Sox18 ctrl kdn HemEC SREBP2 Sox18 GAPDH R+

Figure 3 F Western Blots for full tiffs rev 11/26/24

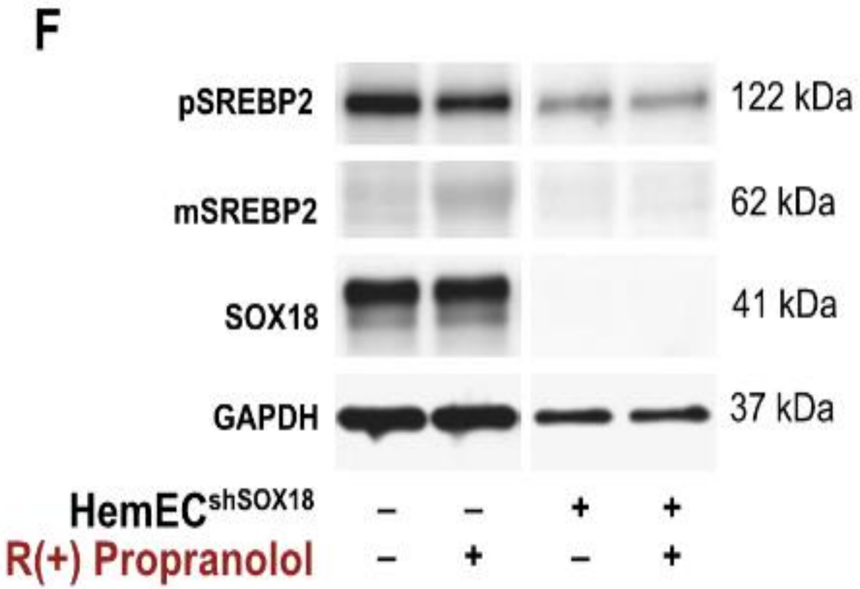

GAPDH

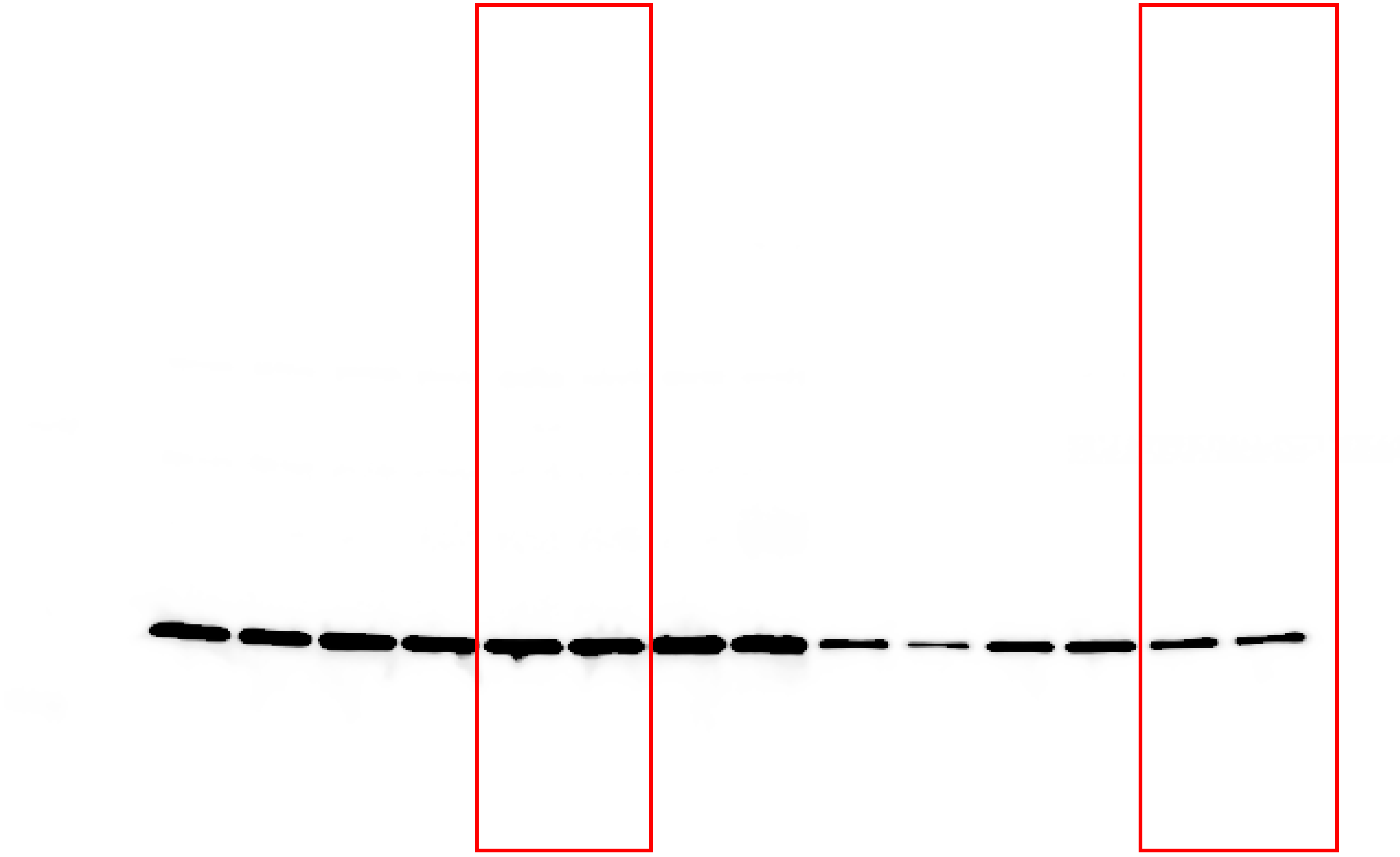

Tiff name: GAPDH threshold affinity autoexposure 23.12.14_16.33.04_PUB_600
Location: G:\Shared drives\SR-Bischoff-Common\Jill Wylie-Sears\For Anne\Figure 3 Find full tiffs 11262024\Figure 3 F Sox18 ctrl kdn HemEC SREBP2 Sox18 GAPDH R+

Figure 3 F Western Blots for full tiffs rev 11/26/24

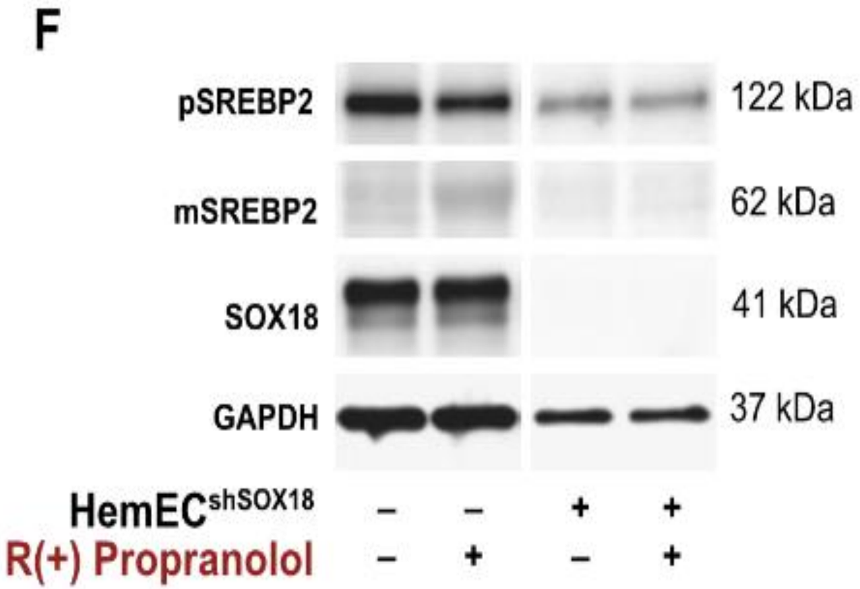

Sox18

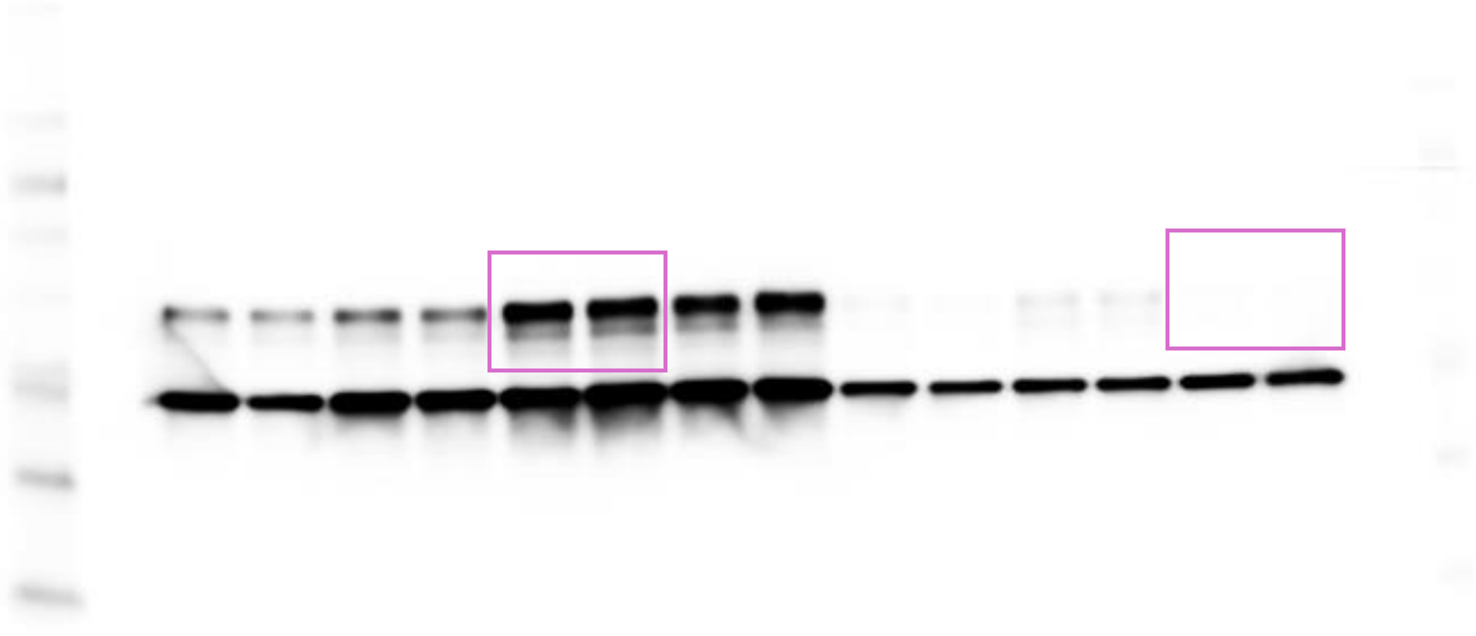

Tiff name: Sox18 GAPDH less gamma 23.12.12_13.30.44_PUB_600
Location: G:\Shared drives\SR-Bischoff-Common\Jill Wylie-Sears\For Anne\Figure 3 Find full tiffs 11262024\Figure 3 F Sox18 ctrl kdn HemEC SREBP2 Sox18 GAPDH R+

SREPBP2 Western Blot HemEC naive and shSOX18 +/- R+ Propranolol 12/13/23

5uM ALLN in RIPA proteinase inhibitors cocktail, No ALLN 2 hrs pre-treatment (following Luke's 4/21/23 protocol)

	1.	2.	3.	4.	5.	6.	7.	8.	9.	10.	11.	12.	13.	14.
PBS	+	-	+	-	+	-	+	-	+	-	+	-	+	-
R+ Propranolol (20 uM)	-	+	-	+	-	+	-	+	-	+	-	+	-	+

Precursor SREBP2
122 kDa

Mature SREBP2
55 - 65 kDa

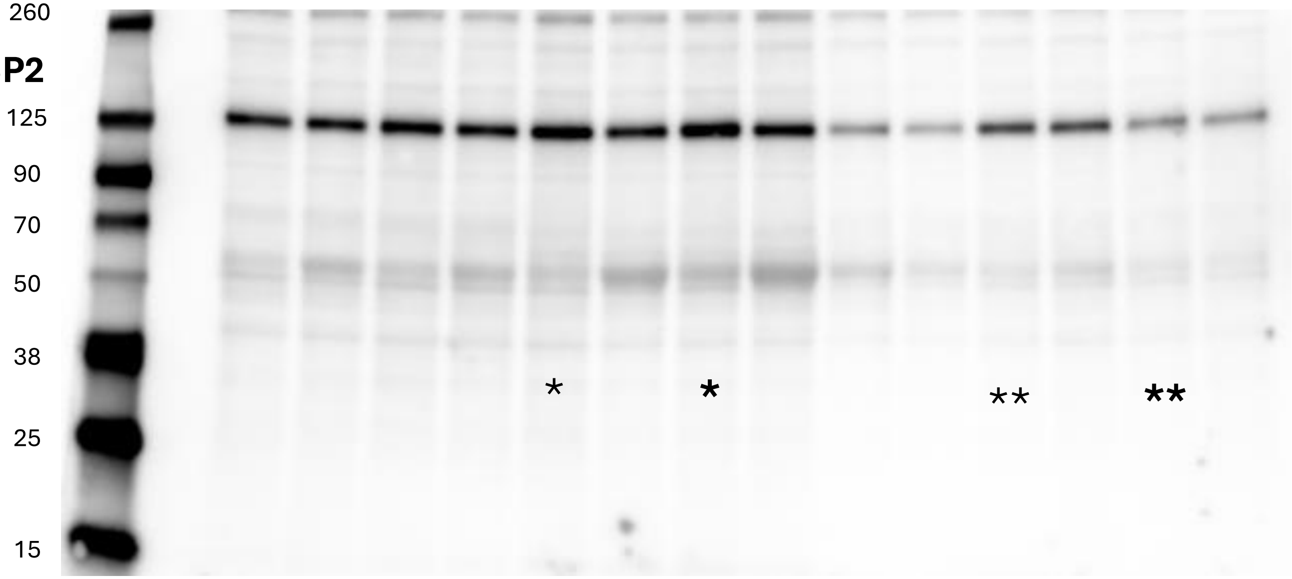

Hemangioma Endothelial Cells

HemEC Sox18 Knockdowns

HemEC 171

* HemEC 171
Naiive (PBS)

<u>Knockdown</u>
<u>HemEC 171</u>

** Sox Kdn
HemEC 171
(PBS)

Overexposure setting Azure Imager

1. HemEC133 PBS
2. HemEC133 R+
3. HemEC150 PBS
4. HemEC150 R+
5. HemEC171 #1 PBS
6. HemEC171 #1 R+
7. HemEC171 #2 PBS
8. HemEC171 #2 R+
9. HemEC133.4 shSOX18 PBS
10. HemEC133.4 shSOX18 R+
11. HemEC150.4 shSOX18 PBS
12. HemEC150.4 shSOX18 R+
13. HemEC171.2 shSOX18 PBS
14. HemEC171.2 shSOX18 R+

All in EGM-2 Complete medium

30 ug protein lysate/well
High MW setting Turbo Transfer
Block 1 hour RT in 5% Milk/TBS-T
Rabbit anti-human SREBP2 (1:125)
Incubation overnight for 23 hours at 4C
2nd rabbit HRP (1:2000)
Incubation overnight (18 hrs) at 4C
All antibodies in 5% Milk/TBS-T
4 ml Clarity Max ECL developer per blot

Sox18, GAPDH Western Blot HemEC naive and shSOX18 +/- R+ Propranolol 12/13/23

	1.	2.	3.	4.	5.	6.	7.	8.	9.	10.	11.	12.	13.	14.
PBS	+	-	+	-	+	-	+	-	+	-	+	-	+	-
R+ Propranolol (20 uM)	-	+	-	+	-	+	-	+	-	+	-	+	-	+

260
125
90
70
SOX18 41 bp
50
38
GAPD H 37 bp
25
15

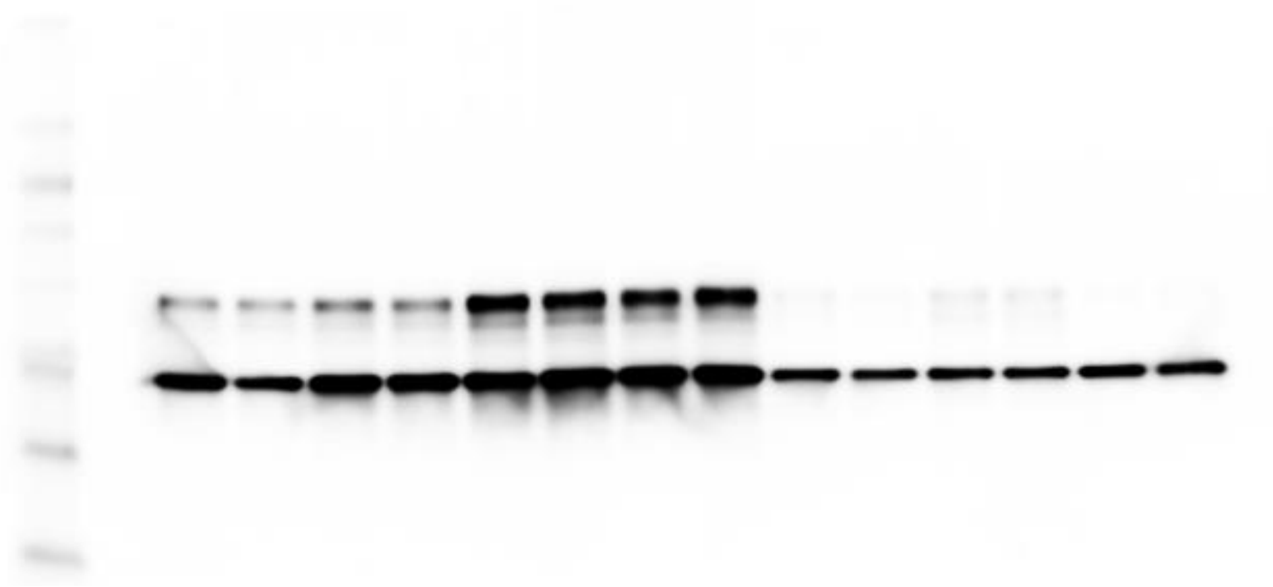

30 sec setting Azure Imager

1. HemEC133 naïve PBS
2. HemEC133 naïve R+
3. HemEC150 naïve PBS
4. HemEC150 naïve R+
5. HemEC171 naïve #1 PBS
6. HemEC171 naïve #1 R+
7. HemEC171 naïve #2 PBS
8. HemEC171 naïve #2 R+
9. HemEC133.4 shSOX18 PBS
10. HemEC133.4 shSOX18 R+
11. HemEC150.4 shSOX18 PBS
12. HemEC150.4 shSOX18 R+
13. HemEC171.2 shSOX18 PBS
14. HemEC171.2 shSOX18 R+

15 ug protein lysate/well
Mixed MW setting Turbo Transfer
Block 1 hour RT in 5% Milk/TBS-T
Mouse anti-human Sox18 (1:500)
Rabbit anti-human GAPDH (1:2500)
Incubation overnight at 4C
2nd mouse HRP (1:2000)
2nd rabbit HRP (1:6000)
Incubation 1 hour at room temperature
All antibodies in 5% Milk/TBS-T
3 ml Clarity Max ECL developer per blot

<u>GAPDH following SREPBP2</u> Western Blot HemEC naive and shSOX18 +/- R+ Propranolol 12/13/23

5uM ALLN in RIPA proteinase inhibitors cocktail, No ALLN 2 hrs pre-treatment (following Luke's 4/21/23 protocol)

	1.	2.	3.	4.	5.	6.	7.	8.	9.	10.	11.	12.	13.	14.
PBS	+	-	+	-	+	-	+	-	+	-	+	-	+	-
R+ Propranolol (20 uM)	-	+	-	+	-	+	-	+	-	+	-	+	-	+

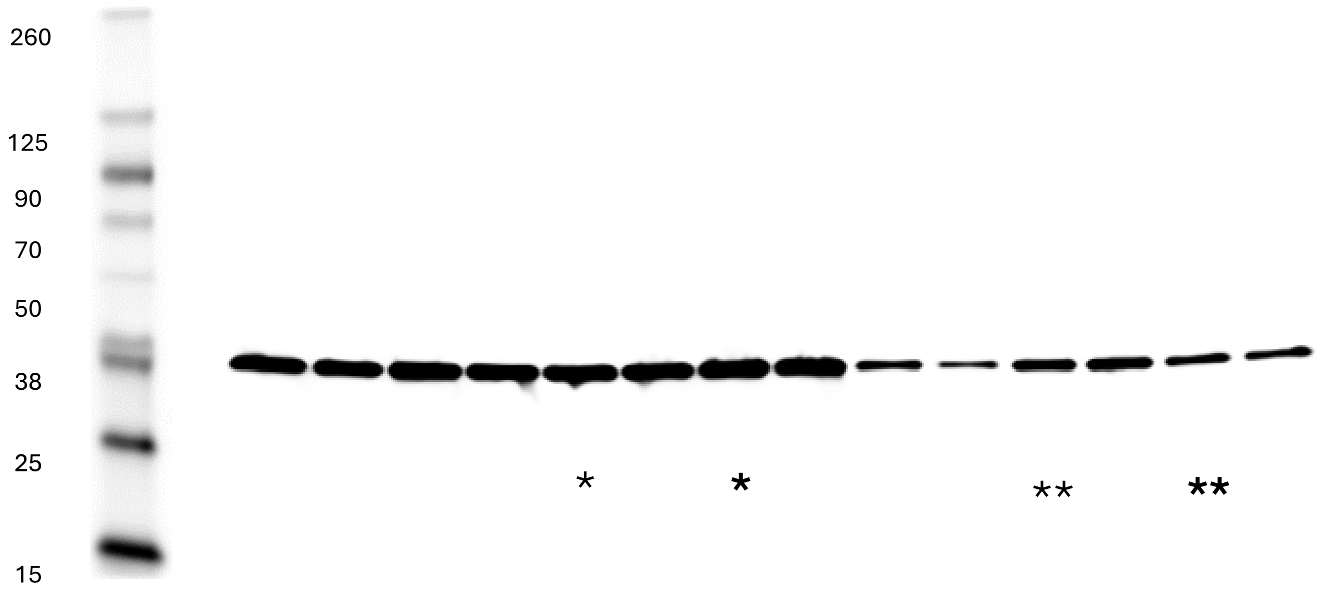

GAPDH
37 kDa

Hemangioma Endothelial Cells

HemEC 171

* HemEC 171
Naiive (PBS)

HemEC Sox18 Knockdowns

<u>Knockdown HemEC 171</u>

** Sox Kdn
HemEC 171
(PBS)

Autoexposure Azure Imager

1. HemEC133 PBS
2. HemEC133 R+
3. HemEC150 PBS
4. HemEC150 R+
5. HemEC171 #1 PBS
6. HemEC171 #1 R+
7. HemEC171 #2 PBS
8. HemEC171 #2 R+
9. HemEC133.4 shSOX18 PBS
10. HemEC133.4 shSOX18 R+
11. HemEC150.4 shSOX18 PBS
12. HemEC150.4 shSOX18 R+
13. HemEC171.2 shSOX18 PBS
14. HemEC171.2 shSOX18 R+

All in EGM-2 Complete medium

30 ug protein lysate/well
High MW setting Turbo Transfer
Block 1 hour RT in 5% Milk/TBS-T
Rabbit anti-human SREBP2 (1:125)
Incubation overnight for 23 hours at 4C
2nd rabbit HRP (1:2000)
Incubation overnight (18 hrs) at 4C
All antibodies in 5% Milk/TBS-T
4 ml Clarity Max ECL developer per blot
